# Phenotypic diversity of CTCs and tdEVs in liquid biopsies of tumour-draining veins is linked to poor prognosis in colorectal cancer

**DOI:** 10.1186/s13046-024-03259-6

**Published:** 2025-01-08

**Authors:** Stefan A. Cieslik, Andrés G. Zafra, Christiane Driemel, Monica Sudarsanam, Jan-Philipp Cieslik, Georg Flügen, Levent Dizdar, Andreas Krieg, Sascha Vaghiri, Hany Ashmawy, Stephen Fung, Miriam Wilms, Leon W. M. M. Terstappen, Afroditi Nanou, Hans Neubauer, Nuh N. Rahbari, Wolfram T. Knoefel, Nikolas H. Stoecklein, Rui P. L. Neves

**Affiliations:** 1 Department of General, Visceral and Pediatric Surgery, University Hospital and Medical Faculty of Heinrich-Heine University Düsseldorf, Moorenstr. 5, 40225 Düsseldorf, Germany; 2 Department of Obstetrics and Gynecology, University Hospital and Medical Faculty of the Heinrich-Heine University Düsseldorf, Moorenstr. 5, 40225 Düsseldorf, Germany; 3 Department of General and Visceral Surgery, Thoracic Surgery and Proctology, Medical Campus OWL, University Hospital Herford, Ruhr University Bochum, 32049 Herford, Germany; 4 Department of Medical Cell BioPhysics, Faculty of Science and Technology, University of Twente, Enschede, 7522 NH The Netherlands; 5 Decisive Science, Amsterdam, The Netherlands; 6 Department of General and Visceral Surgery, University Hospital Ulm, Albert-Einstein-Allee 23, 89081 Ulm, Germany

**Keywords:** Circulating tumour cells, CTCs, Tumour-derived extracellular vesicles, tdEVs, Colorectal cancer, CRC, Tumour-draining vein, Intraoperative blood sampling, CellSearch, Diversity, Single cell analysis

## Abstract

**Background:**

Circulating tumour cells (CTCs) and tumour-derived extracellular vesicles (tdEVs) have great potential for monitoring therapy response and early detection of tumour relapse, facilitating personalized adjuvant therapeutic strategies. However, their low abundance in peripheral blood limits their informative value. In this study, we explored the presence of CTCs and tdEVs collected intraoperatively from a tumour-draining vein (DV) and via a central venous catheter (CVC) prior to tumour resection.

**Methods:**

CellSearch analyses of 395 blood samples from 306 patients with gastrointestinal tumours and 93 blood samples from healthy donors were used to establish and validate gates for the automated detection of CTCs and tdEVs with ACCEPT software and R scripts. The selected gate settings were applied to 227 samples of 142 patients with colorectal cancer (CRC) from two independent collectives. Phenotypic features were obtained via numeric analysis of their fluorescence signals (e.g. size, shape, and intensity) and were used for calculating diversity using Shannon index (SI) of clusters generated via the k-means algorithm after Uniform Manifold Approximation and Projection (UMAP) pre-processing, and standard deviation (SD).

**Results:**

CTCs and tdEVs were more abundant in the DV samples compared to CVC samples (*p* < 0.05). tdEVs were detected in higher numbers than CTCs in both compartments. Importantly, tdEVs in CVCs were associated with tumor spread, whereas CTCs in DVs were linked to tumor size. In both compartments, the prognostic value of tdEVs for overall survival (OS) surpassed that of CTCs, as demonstrated by univariate, multivariate, and Kaplan-Meier analyses. CTCs and tdEVs in DVs were phenotypically distinct, being larger, more eccentric, and displaying stronger cytokeratin intensities (*p* < 0.05) compared to those in CVC samples. Furthermore, increased diversity in CTC and tdEV phenotypes was significantly associated with shorter survival, validating the prognostic relevance of the SD-diversity metric.

**Conclusion:**

Our study demonstrates that DV sampling significantly enhances the detection of prognostically relevant CTCs and tdEVs in CRC patients, underscoring the superior prognostic significance of tdEVs compared to CTCs. Importantly, the combined phenotypic diversity of both markers emerges as a more powerful biomarker than their enumeration alone. These findings suggest that comprehensive, automated analysis of CTCs and tdEVs in DVs may open new avenues for tailoring individualized therapies in CRC patients.

**Supplementary Information:**

The online version contains supplementary material available at 10.1186/s13046-024-03259-6.

## Introduction

Approximately 70% of patients diagnosed with colorectal cancer (CRC) have a local or regional disease (AJCC Stage I-III). For this group of patients, surgical resection is the primary treatment with curative intent and the 5-year survival rate ranges from 91% (local disease) to 73% (regional disease) [1]. However, even after successful tumour resection, approximately 20% of patients experience metastatic relapse within five years [2–4] and in such cases, the 5-year survival rate drastically decreases to only 14% [1]. Identifying the patients at a higher risk of relapse after surgery could improve clinical management and aid in tailoring adjuvant therapeutic strategies.

The molecular mechanisms driving disease progression and relapse are not yet fully understood. Nonetheless, it is recognized that circulating tumour cells (CTCs), as cells that left the primary tumour and retain the potential to seed metastases, play a crucial role in tumour spread [5, 6]. As a result, CTCs have emerged as a promising prognostic tumour biomarker for a variety of malignancies including CRC [7]. CTCs can be efficiently detected via the FDA-cleared CellSearch system, which defines them as EpCAM+/CK+/DAPI+/CD45- cells [8, 9], for which accuracy, precision, linearity, reproducibility and specificity, have been previously described [8–15]. In advanced CRC, approximately 30% of the patients have more than three CellSearch CTCs (CS-CTCs) (in 7.5 mL of blood) and this is associated with poorer clinical outcomes (10, 16–17). In patients with operable CRC, the lower counts and detection rates limit the value of CS-CTCs as biomarker and contribute to conflicting data regarding their prognostic utility [18–22]. In this group of patients, sampling of the tumour or metastasis outflow can increase CS-CTC detection [23–30], and the presence of CS-CTCs in the tumour-proximal mesenteric vein correlates with the serological biomarker CA19-9 [26], but the prognostic value of such cells has not yet been clearly demonstrated [23, 25].

CTC identification with the CellSearch system has some inherent subjectivity. To further enhance standardization of CTC identification in the CellSearch assay, the ACCEPT tool was developed [31, 32]. This tool analyses the raw CellSearch images, segments all objects, quantifies multiple fluorescent parameters, and enables users to objectively discriminate the cells or particles of interest via the quantified parameters. In addition to CTCs, the ACCEPT tool can be used to identify large (> 1 μm) tumour-derived extracellular vesicles (tdEVs), which are co-enriched in CellSearch and defined as EpCAM+/CK+/DAPI-/CD45- particles [33, 34]. tdEVs are important mediators of intercellular communication in both physiological and pathological processes, including cancer [35, 36]. In the peripheral circulation of patients with metastatic CRC, CellSearch tdEVs (CS-tdEVs) are significantly more abundant than CTCs, and their abundance is an independent risk factor for shorter overall survival (OS), suggesting a greater potential as biomarker [37, 38]. However, the presence and clinical relevance of CS-tdEVs in tumour-proximal veins in operable CRC has not yet been explored.

Sampling blood from tumour-proximal veins intraoperatively presents a unique opportunity to improve CTC recovery, and we hypothesize that the same applies to tdEVs. Using the CellSearch and the ACCEPT tool, we analysed CS-CTCs and CS-tdEVs in tumour-proximal blood collected prior to CRC tumour resection to explore the prognostic potential of these two biomarkers for the adjuvant period. Previous studies have shown that the phenotypic heterogeneity of peripheral vein CTCs can be pictured by the CellSearch system and quantified via the ACCEPT tool, and suggest that diversity during patient follow-up could inform on disease progression [39, 40]. Considering that tdEVs are also a diverse class of particles [41], in the present work we investigated the phenotypic heterogeneity of CS-tdEVs and CS-CTCs and its clinical relevance.

## Methods

### Patients and processing of blood samples

A total of 510 samples from 364 patients with gastrointestinal tumours were used in this study (Suppl. Table 1). These included patients admitted at the Department of General, Visceral and Paediatric Surgery, University Hospital of the Heinrich-Heine University Duesseldorf (DU cohort), and a cohort of patients admitted at the Department of General, Visceral and Transplantation Surgery, Heidelberg University Hospital, which has been previously described (HE-cohort) [23]. All patients were admitted for surgery with tumour curative intention. The study was carried out in accordance with Good Clinical Practice guidelines and the Declaration of Helsinki and was independently approved by the Ethics Committees of the Medical Faculty of the Heinrich-Heine University Duesseldorf and the Ruprecht-Karls University Heidelberg. All patients provided written informed consent prior to sample collection. For prognostic analyses in CRC, we used a sub-collective of patients where successful surgical resection of the tumour margins was achieved (R0 CRC) (Suppl. Table 3). From these patients, blood samples from the tumour draining vein (DV, DU-cohort *N* = 76, HE-cohort *N* = 58) were collected intra-operatively prior to tumour resection by puncturing the colic and mesenterico-portal vessels. The blood was collected initially into a syringe and then immediately transferred into CellSave tubes (Menarini, Bologna) for cell preservation. Samples from the central vein catheter (CVC, DU- cohort *N* = 36, HE-cohort *N* = 57) were collected via a central line (either in the internal jugular or subclavian vein) directly into CellSave tubes. All blood samples were processed using the CellSearch Circulating Tumour Cell Kit (Menarini) in the CellSearch AutoPrep system (Menarini) according to the manufacturer’s protocol. Additionally, we used ACCEPT data from 93 healthy volunteers from the IMMC06 clinical trial (NCT00133913) [8, 37].

### Identification of CTCs and tdEVs

The enumeration of CTCs was initially performed manually according to the CellSearch protocol by trained operators. The digitally stored CellSearch image files were subsequently re-analysed with the ACCEPT tool for the automated identification of CTCs (CK+/DAPI+/CD45-), and tdEVs (CK+/DAPI-/CD45-) [42], using the set of gates previously described [34] (Suppl. Table 5). For the present work, we defined three additional selection criteria: one directly in the ACCEPT tool (Eccentricity < 0.9); and two upon downstream analysis of the ACCEPT-output tabular data with an R-based in-house developed script (CK size > DAPI size; CK mean intensity > DAPI mean intensity, or CK mean intensity > 150) (Suppl. Table 5). We validated these criteria in the GI and healthy collectives (Suppl. Methods). In addition ACCEPT was also used to identify white blood cells (WBCs, CK-/DAPI+/CD45+), lymphocyte-derived extracellular vesicles (ldEVs, (CK-/DAPI-/CD45+), and bare nuclei (CK-/DAPI+/CD45-). As part of the standard CellSearch Circulating Tumour Cell assay, samples were scanned for fluorescence in DiOC channel (the 4th channel of the system to detect FiTC) despite the fact that no marker was used for that channel. Fluorescence in the DiOC channel was used to exclude events with high autofluorescence (Suppl. Table 5).

### Calculation of diversity

Diversity was calculated considering all tdEV and CTC events within each cartridge via two different approaches: a cohort-dependent (referred to as the Shannon diversity index) and cohort-independent (referred to as the SD-diversity index).

For Shannon diversity, all nine CK-PE and nine DAPI fluorescence-based parameters quantified by ACCEPT plus the DAPI overlay with CK parameter were considered. ACCEPT data were normalized using min-max normalization and further processed through Uniform Manifold Approximation and Projection (UMAP). UMAP was executed in Python 3.11.5 with the packages “umap-learn” 0.5.3 and “plotly” 5.15.0 and the following settings: “random_state” of 42, “n_neighbors” of 45, “n_components” of 18, “min_dist” of 0.0, and the Euclidean metric. Subsequently, k-means was applied considering different numbers of expected clusters, and Shannon diversity index was calculated for each cartridge on the basis of the distribution of particles through each cluster definition. Following multiple tests on, the distribution across 18 clusters was chosen. For SD-diversity, six CK-PE and six DAPI fluorescence-based parameters quantified by ACCEPT plus the DAPI overlay with CK were considered. All values of the 13 parameters were standardized via z-score normalization, and these normalized values were transformed into their absolute values. For each cartridge individually, we calculated the standard deviation (SD) for each of the 13 parameters, and finally we calculated the mean value of the 13 SD values. The resulting value constituted the SD-diversity index value for the respective cartridge.

## Results

### CTC detection rates increase in DV blood samples

**Fig. 1:**
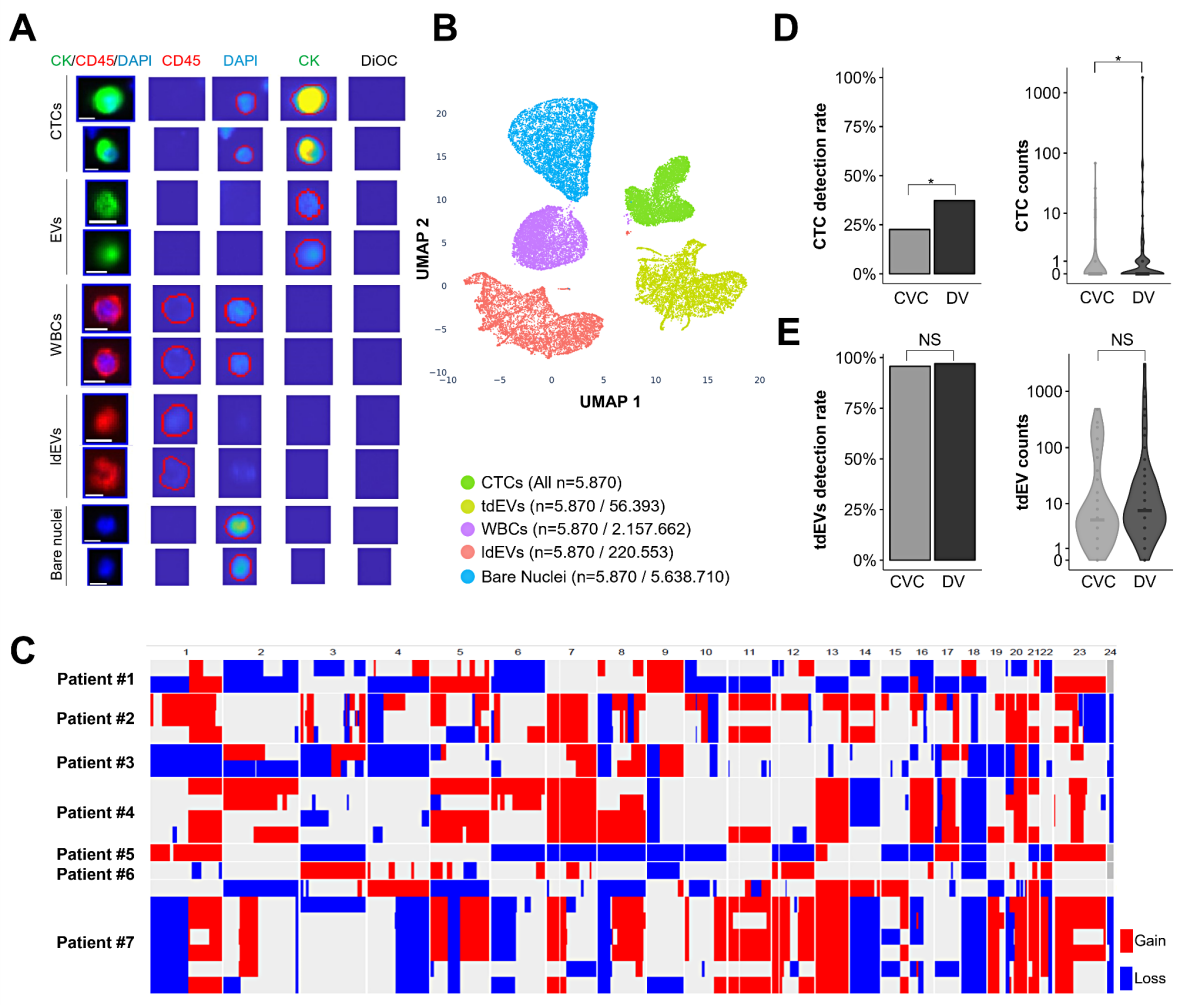
Identification of CTCs and tdEVs with the CellSearch system. **(A)** Representative circulating tumour cells (CTCs), tumour-derived extracellular vesicles (tdEVs), white blood cells (WBCs), lymphocyte-derived extracellular vesicles (ldEVs) and bare nuclei identified using the ACCEPT tool. For every object, a thumbnail image overlay of the three fluorescent channels (CD45, DAPI, PE) and each channel separately is shown. The red contour indicates the detected boundary of each object. The scale bar represents 10 pixels equivalent to 6.4 μm. **(B)** UMAP visualization of the different types of events identified with ACCEPT in samples from CRC R0 patients from the combined DU + HE cohort (*N* = 93 CVCs; *N* = 134 DVs). All detected CTCs (*N* = 5870) are represented, while for purposes of better visualization, for all other populations, we randomly selected *N* = 5870 objects. **(C)** Heatmap representation of chromosomal copy number alterations (CNAs) of 20 single CTCs obtained by low-pass NGS after single-cell whole-genome amplification. **(D)** Detection rates (positivity rates) and counts of CTCs and **(E)** tdEVs in samples from CRC R0 patients from the combined DU + HE cohort (*N* = 93 CVCs; *N* = 134 DVs). The horizontal lines represent the median. *p < 0.05

Our initial aim was to test the hypothesis that the detection frequency of both CTCs and tdEVs can be increased by DV blood sampling. For a more systematic and unbiased assessment of the particles enriched with CS, we used the ACCEPT tool. Using previously defined gates and a set of parameters that we re-defined and validated for different cancer entities (Suppl. Methods, Suppl. Figure 1), we analysed 93 CVC and 134 DV samples from R0 CRC patients (Suppl. Table 3). The set of gates resulted in five groups of particles clearly distinguishable visually, and upon dimensional reduction of data in a UMAP plot: CTCs and tdEVs, as well as co-enriched white blood cells (WBCs), lymphocyte-derived extracellular vesicles (ldEVs) and bare nuclei (Fig. 1A and B). In CVC samples, the CTC positivity rate of 22.6% was perfectly in line with previous studies in M0 CRC patients analysed with CS (average 23%). In DV samples, we indeed observed significantly higher CTC detection rates and counts compared to CVC samples (detection rate: 37.3% vs. 22.6%, *p* = 0.0271; mean count: 16 vs. 2, *p* = 0.0423; range: 0-1790 vs. 0–68) (Fig. 1D). When we investigated the subgroup of M0 patients (*n* = 118) we found a similar distribution, but the differences did not reach statistical significance (detection rate: 35.1% vs. 23.5%, *p* = 0.1100; mean count: 19 vs. 2, *p* = 0.1287; range: 0-1790 vs. 0–68) (Suppl. Figure 2 A). To confirm the malignant nature of the CVC-derived CTCs and the more frequent DV-derived CTCs, we successfully performed low-pass WGS in 20 single CS-CTCs (from six DV samples and one CVC sample) and detected copy number aberrations characteristic of CRC (Fig. 1C). In line with previous reports, we detected tdEVs in greater numbers compared to CTCs (Fig. 1E). Strikingly, as for CTCs, higher tdEV counts were detected in DV samples (median: 8; mean: 89; range = 0-3108) when compared to CVC samples (median: 5; mean: 43; range: 0-492), but these differences did not reach statistical significance (*p* = 0.065) (Fig. 1E). For the M0-subgroup similar results were obtained (Suppl. Figure 2B). Notably, the numbers of CTCs and tdEVs were positively correlated (Suppl. Figure 3).

### Differential association of CTCs and tdEVs from DV and CVC with clinicopathological parameters

Next, we investigated the association of CTCs and tdEVs with clinicopathological parameters (Suppl. Figure 4). Independent of the sampling site, tdEV counts were stronger associated with clinical parameters than CTC counts. In the DV, tdEV counts were strongly associated with higher pT stages (*p* = 0.0008 by Mann-Whitney U test; ρ = 0.332 and *p* < 0.0001 by Spearman correlation analyses) and, to a lesser extent, also with pN stage (*p* = 0.0232 by Mann-Whitney U test; ρ = 0.231 and *p* = 0.0071 by Spearman correlation) (Suppl. Figure 4). Notably, the CTC counts in the DV were also significantly associated with the pT stage (ρ = 0.226 and *p* = 0.0087 by Spearman correlation) (Suppl. Figure 4), suggesting that the DV sampling site more accurately reflects the status of the primary lesion than the CVC site. This positive correlation between pT stage and DV-tdEVs was likewise observed in the M0-subroup (Suppl. Figure 5).

### tdEVs in DV have high prognostic value

**Fig. 2:**
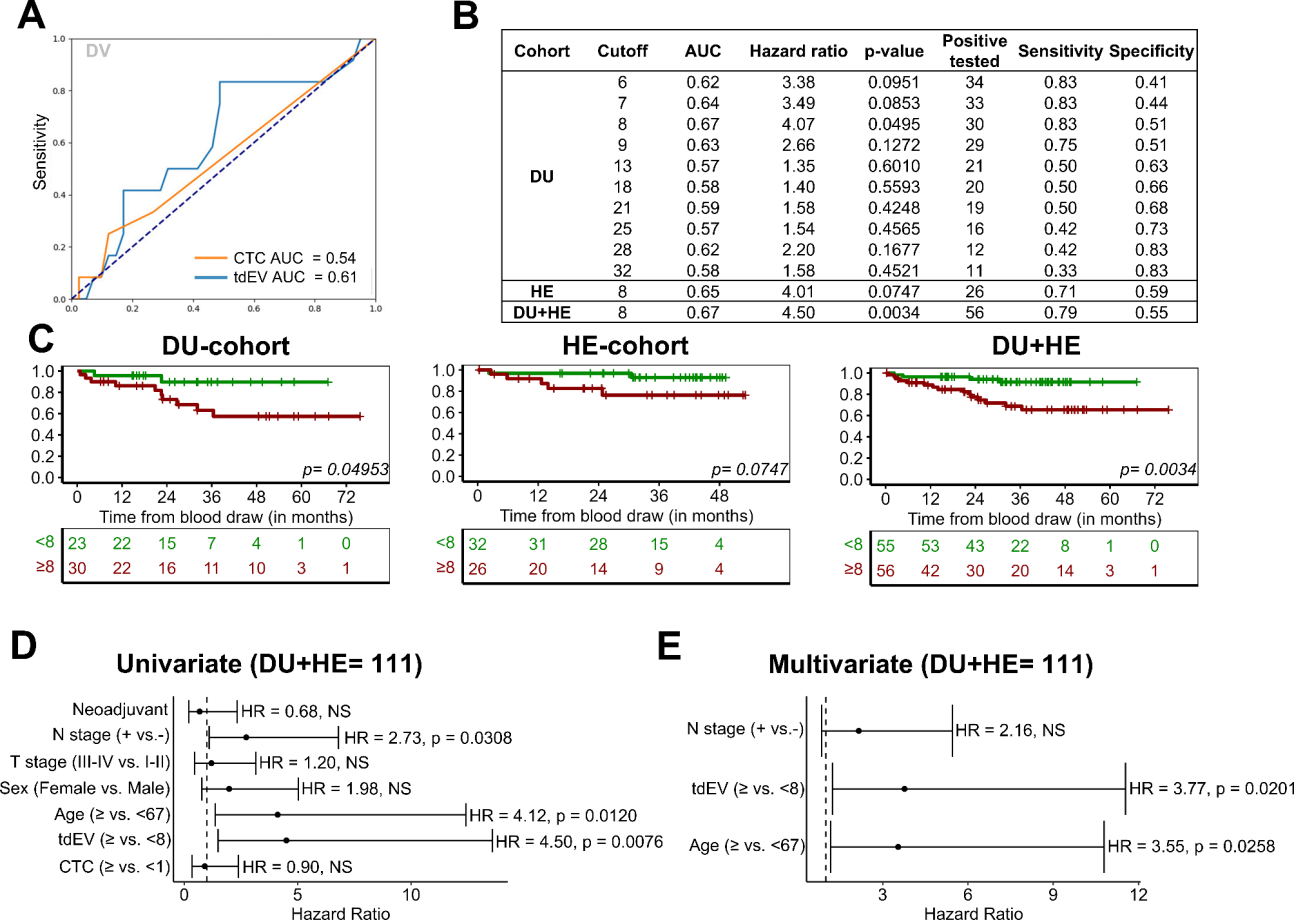
Prognostic value of CS-tdEVs detected in DV samples of CRC R0 M0 patients. **(A)** Receiver operating characteristic (ROC) curves for CTCs and tdEVs and the respective area under the curve (AUC) values calculated for CTCs and tdEVs detected in DV samples of patients from the DU-cohort (*N* = 53). **(B)** Ten most relevant tdEV cut-offs identified in the DV samples from CRC R0 M0 patients of the DU-cohort (*N* = 53), and validation of the 8 tdEV cut-off in the HE (*N* = 58) and DU + HE (*N* = 111) cohorts. **(C)** Kaplan-Meier estimates of overall survival for patients dichotomized on the basis of the 8 tdEV cut-off in the DU (*N* = 53), HE (*N* = 58), and DU + HE (*N* = 111) cohorts of patients. **(D)** Univariate analysis of clinicopathological factors (including the ≥ 8 tdEV cut-off) in the DU + HE cohort of patients (*N* = 111). **(E)** Multivariate analysis of clinicopathological factors, including the ≥ 8 tdEV cut-off, in the DU + HE cohort of patients (*N* = 111)

In order to evaluate the clinical relevance of CTCs and tdEVs we investigated their prognostic impact. For this we focused only on the R0 M0 patients (UICC I-III) and analysed initially a test cohort (DU-cohort) with 60 patients (DV = 53 samples, CVC = 28 samples). First, we determined the best cut-off considering both hazard ratios and AUC values collectively, and identified ≥ 8 tdEVs per sample as the optimal cut-off (HR = 4.07, AUC = 0.67) (Fig. 2B-C). Notably, we generally observed a superior prognostic accuracy of tdEVs for OS compared with that of CTCs, as reflected by their higher area under the curve (AUC) upon receiver operating characteristic (ROC) analysis (Fig. 2A). As expected, elevating the tdEV cut-off value increased the specificity while decreasing the sensitivity. Further validation of the prognostic value of the tdEV cut-off was sought through analysis of DV samples from an independent cohort (HE-cohort, *n* = 58) and the combination of both DU- and HE- cohorts. Strikingly, we could validate this cut-off in the combined DU + HE cohort of 111 DV samples (HR = 4.50, AUC = 0.67, log rank *p* = 0.0034, NPV = 93%), and the cut-off demonstrated a result close to statistical significance in the HE-cohort (HR = 4.01, AUC = 0.65, *p* = 0.0747) (Fig. 2B and D). Furthermore, in the DU + HE cohort, the negative impact of ≥ 8 DV-tdEVs on survival was independent from other parameters according to uni- and multivariate analyses (HR = 3.77, *p* = 0.0201) (Fig. 2E). In CVC samples, ≥ 8 tdEVs lost its discriminatory power. In these samples, the cut-off of ≥ 4 tdEVs had significant prognostic value in the DU-cohort, but it could not be validated in the HE-cohort (Suppl. Figure 6 A). In the case of CTCs, ≥ 1 CTC in DV and CVC was not associated with worse prognosis (Suppl. Figure 6B-D). The prognostic performance of CTCs was inferior to that of tdEVs in the same sampling site (Suppl. Figure 6 A and 6 C). Finally, we investigated the complete DU + HE cohort (M0/M1) and observed similar results using adapted thresholds with stronger significance (Suppl. Figures 7 and 8).

### Phenotypic diversity of tdEVs and CTCs associates with poor survival

Considering the observed phenotypic diversity of CTCs/tdEVs (Fig. 1B), we were interested whether higher diversities in blood samples are associated with a poor prognosis, as previously observed for CTCs in peripheral blood [39, 40, 43–45]. Intriguingly, all phenotypic parameters quantified with ACCEPT were elevated in DVs compared with CVCs, indicating that CTCs and tdEVs from DVs were larger, more eccentric, had stronger CK intensities and, in the case of CTCs, had stronger DAPI signal intensities compared to their CVC counterparts (Suppl. Figures 9–10). In the next step, we assessed the diversity to test its prognostic impact. As an initial strategy, we explored diversity of CTCs and tdEVs by pre-processing the data with UMAP, performing k-means clustering, and calculating the Shannon index (Suppl. Figure 11). As hypothesized, higher diversity strongly correlated with worse prognosis, and we could identify ≥ 0.4214 as a cut-off that significantly dichotomized patients according to OS in the DU-cohort (HR: 3.36, AUC: 0.65), in the HE-cohort (HR: 7.34, AUC: 0.61) and in the DU + HE cohort (HR: 5, AUC: 0.64, log rank *p* = 0.0002, NPV = 88%) (Suppl. Figure 11). Subsequently, for each individual CS sample (cartridge), we calculated a diversity index by averaging the standard deviations of 13 selected parameters across combined tdEVs and CTCs, referred to as the SD-diversity index.

**Fig. 3:**
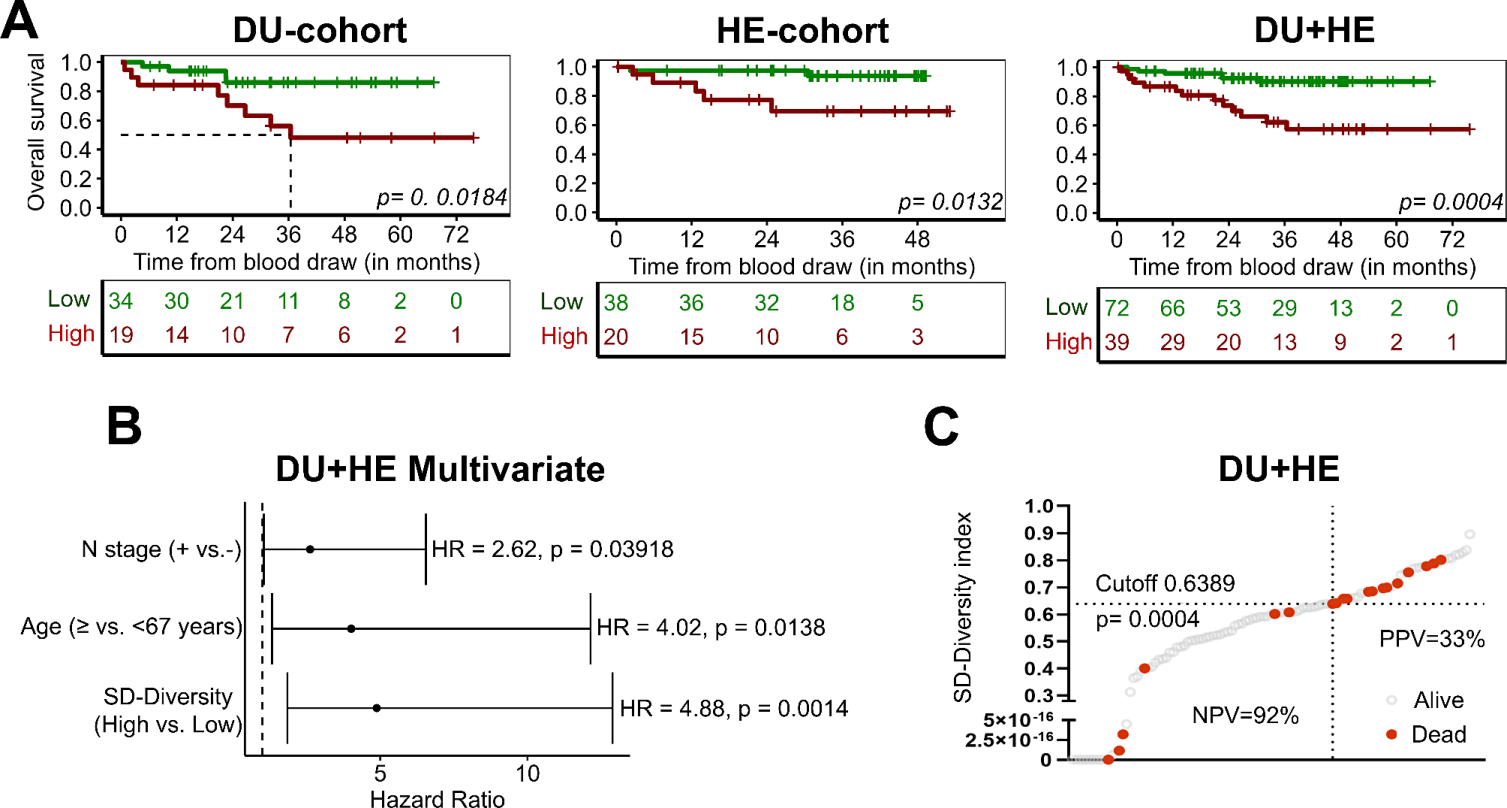
Prognostic value of diversity in DV samples of CRC R0 M0 patients. **(A)** Kaplan‒Meier estimates of overall survival for patients dichotomized on the basis of the SD-diversity cut-off of 0.6389 in the DU (*N* = 53), HE (*N* = 58), and DU + HE (*N* = 111) cohorts of patients. **(B)** Multivariate analysis was performed including variables that were found to be significant in the univariate model. **(C)** SD-diversity index calculated for each of the *N* = 111 patients of the DU + HE cohort showing the positive predictive value (PPV) and negative predictive value (NPV) of the cutoff 0.6389 as a biomarker. In red are indicated the patients that died

Using this approach, higher diversity was strongly associated with worse prognosis. In DV samples from the DU cohort, the optimal patient dichotomization was achieved with an SD-diversity index threshold of ≥ 0.6389 (HR: 3.83, AUC: 0.70). Notably, this cut-off was validated in the HE cohort (HR: 6.20, AUC = 0.71) and the combined DU + HE cohorts (HR = 4.88, AUC = 0.70, log-rank *p* = 0.0004, NPV = 92%) (Fig. 3A and C, Suppl. Figure 12). In addition, the SD-diversity index remained significant in the multivariate model for the combined DU + HE cohort of patients with DV samples (HR: 4.88, *p* = 0.0014) (Fig. 3B), even when the ≥ 8 tdEVs cut-off was included in the model (HR: 3.92, *p* = 0.0457) (Suppl. Figure 12C). Across all three cohorts, the OS predictive value of SD-diversity ≥ 0.6389 was superior to the previously identified tdEV enumeration cut-off (≥ 8 tdEVs) (Suppl. Figure 12A). Notably, applying the same diversity index cut-offs demonstrated a similar prognostic impact in the combined M0/M1 cohort (Suppl. Figures 13–14). Furthermore, higher particle diversity in CVC samples also identified patients with worse prognosis, with the DU cohort cut-offs successfully validated in the HE and combined DU + HE cohorts (data not shown). Finally, to evaluate the robustness of the newly established SD-diversity metric, we aimed to validate the general observation that higher phenotypic SD-diversity correlates with worse survival outcomes, rather than focusing on a specific cut-off. Given the limited availability of DV data from CS, we re-analyzed previously published CS data from peripheral blood samples of a CRC M0 cohort (IMMC-26) with OS and progression-free survival (PFS) data [20]. Although we had to adjust the cut-off for SD-diversity due to differences between peripheral blood and DV samples—likely reflecting the higher diversity in DV samples—SD-diversity remained a significant prognostic biomarker for both OS and PFS, independent of T- and N-stage (Suppl. Figure 15).

## Discussion

Classic clinicopathological parameters have limitations when deciding on adjuvant therapy in primary CRCs. In this context, circulating biomarkers in liquid biopsy samples hold promise for improving clinical decision-making. This study evaluated whether the phenotypic diversity of EpCAM-enriched, cytokeratin-positive objects (i.e., CTCs and tdEVs) in CS images of blood samples can identify operable CRC patients with poor prognosis. Our approach identifies patients with a high diversity of CK-positive objects who are at risk for poor survival outcomes. To conduct this study, we performed two CellSearch assays per patient on two intra-operatively collected blood samples: one from a central venous catheter (CVC) and the other from a tumour-draining vein (DV), located closer to and downstream of the tumour, before the blood passed through the liver.

Our data confirmed previous investigations demonstrating that both CTCs and tdEVs are significantly more prevalent in DV samples [25, 26, 46–53], presumably due to their higher concentration proximal to the tumour, where tumour-derived material invades and leaks directly into the bloodstream. This was further validated by our ACCEPT analysis of a second, independent, and previously published DV collective [23]. The rarity of CTCs in CVC blood together with the observed markedly higher prevalence of biomarkers in DV blood led us to discontinue analysing the CVC samples in the DU cohort.

Importantly, we can make three new vital observations. First, CTCs and tdEVs in DVs have different morphologic features than their CVC sample counterparts. The automated CS image analysis via ACCEPT enabled us to record up to 19 parameters per identified CK-positive object (19 for CTCs and 9 for tdEVs), clearly showing that DV samples were dominated by larger and more intensely CK-stained CTCs and tdEVs. Together with the observed reduction in the frequency of CTCs and tdEVs in CVC blood, these data suggest their arrest during the liver passage [25]. Therefore, it is tempting to speculate that CS analysis captures information on two critical components for hepatic metastasis: CTCs, which are potential precursors of hepatic metastasis [54–56], and tdEVs, which might facilitate metastasis by releasing their cargo to support pre-metastatic niche formation in the liver [57–61].

The second intriguing observation of our study concerns the differential association of tdEV/CTC frequency with tumour stage. While the correlations with pN- and M-stages were similar in DV and CVC samples, the frequencies of CTCs and tdEVs correlated significantly with the pT-stage only in the DV sample. This observation suggests that DV blood can effectively enable the detection of CK-positive tumour material directly released from the primary tumour. This is further supported by the finding that there was a greater likelihood of obtaining genomically aberrant profiles typical for CRC in CTCs isolated from DVs than in CVC samples. Although we did not perform a systematic analysis of the genomic profiles here, it was interesting to observe the CNAs in CS-detected CTCs of non-metastatic CRC, which, to the best of our knowledge, have not been described before, likely owing to their shallow concentration in peripheral blood at this disease stage.

Third, we observed that the prognostic information of DV samples clearly outperformed that of CVC samples. Nevertheless, the prognostic value of CS-CTCs could not be validated in the HE and pooled DU + HE cohorts. We posit that this inconsistency arises most likely from the combination of relatively small patient cohorts and the comparatively low frequency of CS-CTC detection, which, even with tumour-proximal sampling, remains notably lower than in patients with metastatic CRC. Remarkably, none of the previous studies could confirm a prognostic role for CS-CTCs in DV blood of non-metastatic CRC patients. Further research involving larger patient cohorts might be necessary to clarify the prognostic value of CS-CTCs in DV blood. However, our data show that the more abundant tdEVs can be complementary and that there is no need for the exclusive analysis of CS-CTCs. The tdEVs alone were significantly associated with poor survival over a broader range of thresholds, for which the best threshold could be confirmed in the independent HE cohort and in the pooled analysis. Similar to our findings, a previous study described tdEVs in the peripheral blood of patients with metastatic CRC and reported higher OS hazard ratios than CTCs did [38]. The observed prognostic impact, particularly of tdEVs, and the acknowledgment of the morphologic diversity of both CK-positive CS-CTCs and CS-tdEVs prompted our investigation into the prognostic significance of this diversity. Our underlying hypothesis posited that highly invasive and thus more aggressive cancers would exhibit a greater prevalence of CK-positive cells and tdEVs, yielding greater morphologic diversity in the DV sample before hepatocellular transit. As a starting point to test this hypothesis, we used k-means clustering and Shannon diversity index to analyse diversity on the basis of the morphological features of the CK-positive biomarkers, as previously described [39, 44]. In contrast to these previous studies, we pre-processed the initial dataset with UMAP since it was reported to improve the accuracy of subsequent clustering tools, including k-means [62, 63]. Our comprehensive analysis of the phenotypic attributes of both CTCs and tdEVs unveiled substantial diversity within and between DV samples. Interestingly, our data revealed that increased phenotypic diversity of CTCs and tdEVs correlated with adverse prognostic outcomes. Furthermore, we validated the prognostic impact of SD-diversity in a relatively large, independent CS dataset from M0 CRC patients. Given the limited availability of DV data in CRC, we utilized peripheral blood sample data for this purpose. However, the findings confirmed that SD-diversity is not confined to a specific sample type but represents a broader phenomenon, strongly reinforcing our hypothesis with this dataset. In prostate cancer, the diversity in the phenotypic attributes of CS-CTCs, as determined with ACCEPT, has been found to have a significant inverse correlation with progression-free and overall survival. CTC diversity has been associated with the development of therapy resistance [39], and specific morphologic subclasses of CS-CTCs have been linked to more advanced disease stages [43]. In this context, it is worth emphasizing that Scher and colleagues were the first, using the Epic Sciences CTC platform, to investigate the role of the Shannon index as a diversity measure of CTC morphology for predictive purposes. These results indicate a clear association between low CTC phenotypic heterogeneity and improved survival in patients treated with androgen receptor signalling inhibitors. In contrast, high heterogeneity was associated with better OS in patients treated with taxane chemotherapy [44].

Notably, previous works [39, 44] and our approach to describe diversity on the basis of the Shannon index require retrospective data from the complete sample set for cluster definition. This implies that a prospective analysis of individual samples cannot be performed, posing a limitation of this method. Because the number of particles varies greatly between samples, clustering tools and the resulting number of clusters are highly influenced by this factor. Consequently, if such clustering would be performed separately for each individual sample, the basis for calculating diversity would be sample specific, and the calculated value of diversity could not be compared between samples. This motivated us to explore a simpler alternative to measure diversity. As an easily applicable approach, we calculated a diversity index as a mean of the standard deviation of the morphological attributes (SD-diversity index). In contrast to the Shannon index, this index can be applied prospectively to individual blood samples. Importantly, our simplified method confirmed the worse prognosis resulting from high diversity, supporting the use of this method in prospective studies. In metastatic breast cancer patients, the presence of particles belonging to different classes of CTCs and tdEVs, as defined by their expression of CK and HER2 measured with CellSearch and ACCEPT, was linked to poorer clinical outcomes than in patients where only one class of particles was detected [40]. In conclusion, these and other similar observations highlight the added clinical value that can be obtained from CTCs and tdEVs by conducting a more comprehensive characterization beyond simple enumeration.

## Conclusions

In conclusion, our study demonstrates that DV sampling enhances the detection of prognostically relevant CTCs and tdEVs in non-metastatic CRC. Importantly, this work highlights the potential of CTC and EV phenotypic diversity to enhance patient prognostication to potentially improve clinical decision-making for CRC patients in the adjuvant setting. Future confirmatory studies could evaluate our approach for assessing CS-CTC/CS-tdEV diversity in comparison to other minimal residual disease markers, such as ctDNA, in terms of prognostic accuracy and cost-effectiveness. Additionally, investigating whether a combination of these markers could further improve prognostic performance may offer new insights into optimizing patient management strategies.

## Electronic supplementary material

Below is the link to the electronic supplementary material.

Supplementary Material 1

## Data Availability

The datasets used and/or analysed during the current study are available from the corresponding author on reasonable request.

## Abbreviations

CTCs: Circulating tumour cells
tdEVs: Tumour-derived extracellular vesicles
DV: Tumour-draining vein
CVC: Central venous catheter
CRC: Colorectal cancer
SI: Shannon index
SD: Standard deviation
OS: Overall survival
AJCC: American Joint Committee on Cancer
CS-CTCs: CellSearch circulating tumour cells
EpCAM: Epithelial cell adhesion molecule
CK: Cytokeratin
DAPI: − 4’,6-diamidino-2-phenylindole
WGS: Whole-genome sequencing
UMAP: Uniform Manifold Approximation and Projection
AUC: Area under the curve
HR: Hazard ratio
NPV: Negative predictive value
GI: Gastrointestinal
HE: Heidelberg (referring to a specific cohort)
DU: Düsseldorf (referring to a specific cohort)
NPV: Negative predictive value
WBC: White blood cell
ldEV: Lymphocyte-derived extracellular vesicle
