# Supplementary material for: Phenotypic diversity of CTCs and tdEVs in liquid biopsies of tumour-draining veins is linked to poor prognosis in colorectal cancer
Source: J Exp Clin Cancer Res. 2025 Jan 8;44:9. doi: 10.1186/s13046-024-03259-6 (PMC11708080; doi:10.1186/s13046-024-03259-6)
Supplement: Supplementary file 1 — Supplementary Material 1 [file 13046_2024_3259_MOESM1_ESM.docx]

**Supplementary Information**

**Phenotypic diversity of CTCs and tdEVs in liquid biopsies of tumour-draining veins is linked to poor prognosis in colorectal cancer**

Stefan A. Cieslik^1^, Andrés G. Zafra^1^, Christiane Driemel^1^, Monica Sudarsanam^1^, Jan-Philipp Cieslik^2^, Georg Flügen^1^, Levent Dizdar^1^, Andreas Krieg^1,3^, Sascha Vaghiri^1^, Hany Ashmawy^1^, Stephen Fung^1^, Miriam Wilms^1^, Leon W.M.M. Terstappen^1,4,5^, Afroditi Nanou^4^, Hans Neubauer^2^, Nuh N. Rahbari^6^, Wolfram T. Knoefel^1^, Nikolas H. Stoecklein^1*^ and Rui P. L. Neves^1*^

**Affiliations**

^1^ Department of General, Visceral and Pediatric Surgery, University Hospital and Medical Faculty of the Heinrich- Heine- University Düsseldorf, Moorenstr. 5, 40225 Düsseldorf, Germany

^2^ Department of Obstetrics and Gynecology, University Hospital and Medical Faculty of the Heinrich- Heine- University Düsseldorf, Moorenstr. 5, 40225 Düsseldorf, Germany

^3^ Department of General and Visceral Surgery, Thoracic Surgery and Proctology, University Hospital Herford, Medical Campus OWL, Ruhr University Bochum, 32049 Herford, Germany

^4^ Department of Medical Cell BioPhysics, Faculty of Science and Technology, University of Twente, 7522 NH Enschede, The Netherlands

^5^ Decisive Science, Amsterdam, The Netherlands

^6^ Department of General and Visceral Surgery, University Hospital Ulm, Albert-Einstein-Allee 23, 89081 Ulm, Germany

*shared senior authorship

**Table of Contents**

**Supplementary Methods**

**Supplementary References**

**Supplementary Tables (Suppl. Table 1 - 5)**

**Supplementary Figures (Suppl. Figure 1 - 15)**

**Supplementary Methods**

**Validation of ACCEPT criteria for enumeration of CellSearch-CTCs from CRC samples**

For more automated and user-unbiased enumeration of CellSearch-CTCs (CS-CTCs), we employed the ACCEPT tool (Zeune2017). In order to validate the ACCEPT settings previously used for identification of CTCs (ACCEPT-UT settings) (**Suppl. Table 5**) (Nanou2018), we analysed CellSearch results of a total of 395 samples from GI/lung tumour patients and 93 control individuals with ACCEPT and compared the ACCEPT-CTC counts to counts obtained upon manual enumeration, according to the standard CS protocol (**Suppl. Fig. 1**). The described ACCEPT settings (**Suppl. Table 5**) resulted in a substantial increase in the CTC counts compared to those obtained with manual enumeration (**Suppl. Fig. 1**). We noted that this was due to the inclusion of several artefacts which triggered us to adapt the ACCEPT settings to our collective of samples. To the criteria reported previously we added a lower limit for eccentricity in order to exclude events with less round morphologies, and we considered the relation between CK and DAPI signals to exclude events with nucleus area larger than cytoplasm (ACCEPT-DU settings) (**Suppl. Table 5**). Taking the results obtained upon manual CTC enumeration as reference, in the collective of all malignant 362 GI/lung samples the new ACCEPT-DU settings were more specific (92%) and less sensitive (65%) compared to the previous settings (64% and 86%, respectively) (**Suppl. Fig. 1A**). Moreover, the agreement between CTC counts assessed manually and automatically with ACCEPT improved when using the ACCEPT-DU settings compared to the UT settings, as indicated by a higher Cohen’s kappa coefficient (0.59 compared to 0.43, p<0.0001) and Spearman rank-order correlation (0.67 compared to 0.59, p<0.0001) (**Suppl. Fig. 1 A-C**). Furthermore, in samples obtained from healthy donors (N=93), or patients with benign tumours (N=33), the DU settings decreased the positivity rate from 32% to 13%, and from 27% to 12%, respectively, values more in line with the ones obtained manually (3% and 15%, respectively) (**Suppl. Fig. 1 A**). Taken together, the results collected indicate that the ACCEPT-DU settings are more stringent and have a performance more similar to the manual enumeration. This suggests that the new settings can be confidentially used for CTC enumeration in CRC samples.

**Visualization of particle diversity in CellSearch-enriched samples**

Initial visualization of particle diversity in CellSearch cartridges (**Figure 1B**), was based on all features extracted by ACCEPT (total of nine features per fluorescence channel). Uniform Manifold Approximation and Projection (UMAP) was used to reduce data dimensions to two and to plot the data, using the following settings: “random_state” of 42, “n_neighbors” of 30, “n_components” of 2, “min_dist” of 0.1 (0.4 in some cases) and a Euclidean metric. The total number of CTCs detected was plotted (n=5870), but the number of particles belonging to the other groups was limited to that same number of 5870, for purposes of computational efficiency and better visualization. The class of the particles was determined by the gates in ACCEPT (**Suppl. Table 5**), and this was used as a particle attribute to colour the particles in the final UMAP plot.

**Isolation and copy number aberration profiling of single CTCs**

To confirm the malignant nature of the identified CTCs we performed genome-wide profiling of chromosomal copy number aberrations (CNAs). For that, we isolated single DAPIpos/CKpos/CD45neg cells from the CellSearch cartridges by flow cytometry using a MoFlo XDP sorter (Beckman Coulter, Germany) as we previously described (Neves2014). Single cells were sorted into individual empty PCR tubes and stored at -20 °C until analysis.

Whole genome amplification (WGA) was performed using MseI-based adapter-linker PCR as previously described (Klein1999, Stoecklein2002), commercialized as Ampli1TM WGA Kit by Menarini Silicon Biosystems (Bologna, Italy). The quality of the WGA product was evaluated by a control multiplex PCR as previously described (Knijnenburg2007) and good quality was defined as ≥3 signals in the control PCR (Mohlendick2013). Illumina-compatible libraries were prepared from good quality WGA products using Ampli1^TM^ LowPass kit for Illumina (Menarini Silicon Biosystems, Bologna, Italy) according to the manufacturer instructions. For high-throughput and more standardized processing, the manufacturer procedure was implemented in a fully automated workflow on a STARlet Liquid Handling Robot (Hamilton, Reno, NV, USA). Resulting libraries were sequenced on MiSeq or HiSeq instruments (Illumina, Hayward, CA, USA). The WGS-generated sequences were aligned to the human reference genome (hg19) using Burrows-Wheeler Alignment Tool (BWA 0.7.15). CNAs were predicted by using QDNAseq 11.0 with a window size of 500 kb. “Gain” and “loss” calls were filtered out by residual (> 4 standard deviations, SD i.e a default setting) and segmented copy number data of each sample were extracted in log2Ratio values. To assess the quality of the generated profiles, sequence-aligned reads were randomly sub-sampled closer to 200,000 reads and copy number analysis was performed with QDNAseq using a window size of 500 kb. Samples with high Derivative Log Ratio Spread (DLRS >= 0.35) in sub-sampled profiles or samples with high interquartile range (IQR >= 0.35) were excluded from further analyses due to high noise. Samples were classified as aberrant if the whole genome alteration percentage (GAP) was above 2.5%, a threshold defined based on a dataset of WBCs (data not shown).

**Basic data visualization**

We utilized the ggplot2 package (version “3.4.2”) in R to create violin-, bar-, correlation-, ROC- and forest- plots. GraphPad Prism (Version 7.03, GraphPad Software, San Diego, CA, USA) was also used for data visualization.

**Statistical analyses**

All the data was organized and analysed using in-house developed R and Python scripts. The Kolmogorov-Smirnov test was used to confirm non-normal distribution of CTC and tdEV counts across the different subsets of patient samples. Non-parametric two-tailed test (Mann –Whitney U-test), Wilcoxon Signed Rank test, and Chi-Square test were used for computing statistical significance for independent, matched, and collectives with nominal values respectively. In Cox regression analyses, the significant variables from the univariate analysis (p<0.05) were fit in multivariate. Receiver operating characteristic (ROC) curves (considering the variable “dead/alive”), the respective area under the curve (AUC), as well as the sensitivity, specificity and Youden Index values for different cutoffs were determined using R.

**Survival analyses**

A vector file containing all metric values of the respective biomarker was analysed by an in-house R script in a loop to automate the analysis of all possible cutoffs. The packages used were: (i) The 'survival' package (version 3.5-5) to perform survival analysis by calculating relevant prognostic values, including hazard ratio and log-rank p-value. (ii) The 'pROC' package (version 1.18.0) to compute area under the curve (AUC) values. The best cutoff chosen was the one that provided the best combination of the lowest p-value in the log-rank test and the highest AUC value in the ROC analysis. The “survminer” package (version “0.4.9”) was chosen to create Kaplan-Meier curves together with risk tables.

**Supplementary References**

Zeune L, van Dalum G, Decraene C, Proudhon C, Fehm T, Neubauer H, Rack B, Alunni-Fabbroni M, Terstappen LWMM, van Gils SA, Brune C. Quantifying HER-2 expression on circulating tumor cells by ACCEPT. PLoS One. 2017 Oct 30;12(10):e0186562. doi: 10.1371/journal.pone.0186562. PMID: 29084234; PMCID: PMC5662084.

Nanou A, Coumans FAW, van Dalum G, Zeune LL, Dolling D, Onstenk W, Crespo M, Fontes MS, Rescigno P, Fowler G, Flohr P, Brune C, Sleijfer S, de Bono JS, Terstappen LWMM. Circulating tumor cells, tumor-derived extracellular vesicles and plasma cytokeratins in castration-resistant prostate cancer patients. Oncotarget. 2018 Apr 10;9(27):19283-19293. doi: 10.18632/oncotarget.25019. PMID: 29721202; PMCID: PMC5922396.

Nanou A, Zeune LL, Terstappen LWMM. Leukocyte-Derived Extracellular Vesicles in Blood with and without EpCAM Enrichment. Cells. 2019 Aug 20;8(8):937. doi: 10.3390/cells8080937. PMID: 31434250; PMCID: PMC6721753.

Neves RP, Raba K, Schmidt O, Honisch E, Meier-Stiegen F, Behrens B, Möhlendick B, Fehm T, Neubauer H, Klein CA, Polzer B, Sproll C, Fischer JC, Niederacher D, Stoecklein NH. Genomic high-resolution profiling of single CKpos/CD45neg flow-sorting purified circulating tumor cells from patients with metastatic breast cancer. Clin Chem. 2014 Oct;60(10):1290-7. doi: 10.1373/clinchem.2014.222331. PMID: 25267515.

Klein CA, Schmidt-Kittler O, Schardt JA, Pantel K, Speicher MR, Riethmüller G. Comparative genomic hybridization, loss of heterozygosity, and DNA sequence analysis of single cells. Proc Natl Acad Sci U S A. 1999 Apr 13;96(8):4494-9. doi: 10.1073/pnas.96.8.4494. PMID: 10200290; PMCID: PMC16360.

Stoecklein NH, Erbersdobler A, Schmidt-Kittler O, Diebold J, Schardt JA, Izbicki JR, Klein CA. SCOMP is superior to degenerated oligonucleotide primed-polymerase chain reaction for global amplification of minute amounts of DNA from microdissected archival tissue samples. Am J Pathol. 2002 Jul;161(1):43-51. doi: 10.1016/S0002-9440(10)64155-7. Erratum in: Am J Pathol. 2003 Dec;163(6):2645. PMID: 12107088; PMCID: PMC1850686.

Knijnenburg J, van der Burg M, Tanke HJ, Szuhai K. Optimized amplification and fluorescent labeling of small cell samples for genomic array-CGH. Cytometry A. 2007 Aug;71(8):585-91. doi: 10.1002/cyto.a.20412. PMID: 17458882.

Möhlendick B, Bartenhagen C, Behrens B, Honisch E, Raba K, Knoefel WT, Stoecklein NH. A robust method to analyze copy number alterations of less than 100 kb in single cells using oligonucleotide array CGH. PLoS One. 2013 Jun 25;8(6):e67031. doi: 10.1371/journal.pone.0067031. PMID: 23825608; PMCID: PMC3692546.

**Supplementary Table 1** – Overview of all samples used in this study.

**
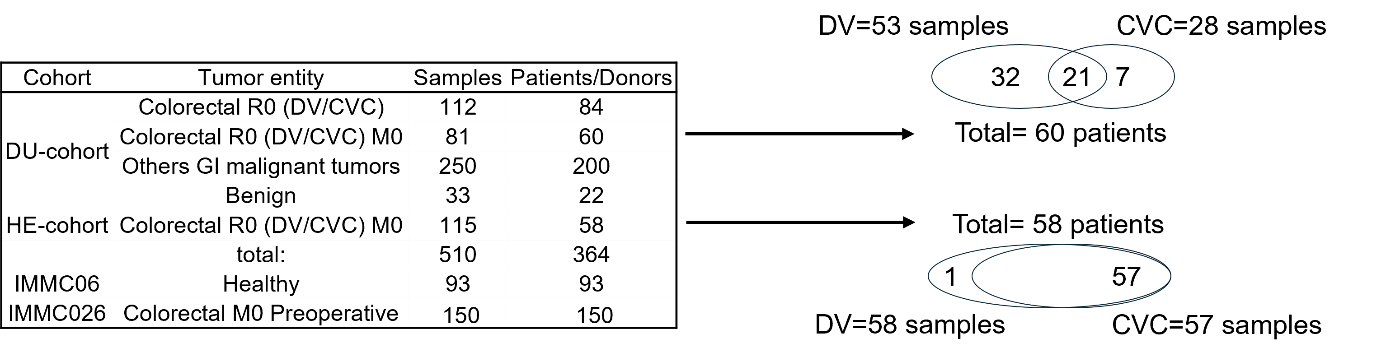
**

R0 - cases where successful surgical resection of the tumour margins was achieved; DV - draining vein; CVC - central venous catheter; GI - gastrointestinal

**Supplementary Table 2** – ICD-O information on the samples used for the gate validation.

| **ICDO Classification** | **ICDO Description** | **Sample**  **Number** |
| --- | --- | --- |
| C18; C19; C20 | Colorectal (R1) | 86 |
| C18; C19; C20 | Colorectal (Not Primary; Metastases) | 23 |
| C15 | Malignant neoplasm of the esophagus | 9 |
| C16 | Malignant neoplasm of the stomach | 25 |
| C17 | Malignant neoplasm of the small intestine | 2 |
| C22 | Malignant neoplasm of the liver and intrahepatic bile ducts | 8 |
| C24 | Malignant neoplasm of other and unspecified parts of the biliary tract | 5 |
| C25 | Malignant neoplasm of the pancreas | 81 |
| C34 | Malignant neoplasm of the bronchus and lung | 11 |
|  | **Total Other Cancer Entities:** | **250** |
| D12.2 | Benign neoplasm of the ascending colon | 2 |
| D12.5 | Benign neoplasm of the sigmoid colon | 1 |
| D12.7 | Benign neoplasm of the rectum | 1 |
| D13.5 | Benign neoplasm of the pancreas | 2 |
| D13.6 | Benign neoplasm of the extrahepatic bile ducts | 7 |
| D13.7 | Benign neoplasm of the liver and intrahepatic bile ducts | 2 |
| D37.7 | Neoplasm of uncertain or unknown behavior of the endocrine glands | 7 |
| K85.9 | Acute pancreatitis, unspecified | 2 |
| K86.0 | Alcohol-induced chronic pancreatitis | 5 |
| K86.1 | Other chronic pancreatitis | 3 |
| K86.2 | Cyst of pancreas | 1 |
|  | **Total Benign:** | **33** |

**Supplementary Table 3** – Clinicopathological information on the patients with colorectal cancer from the DU- and HE-cohorts where successful surgical resection of the tumour margins was achieved (R0) and from whom samples were collected intraoperatively from the draining vein (DV) and/or the central venous catheter (CVC).

|  |  | **DV=134 samples** | | **CVC = 93 samples** | | |
| --- | --- | --- | --- | --- | --- | --- |
|  |  | **DU-cohort**  **DV N=76** | **HE-cohort**  **DV N=58** | **DU-cohort**  **CVC N=36** | **HE-cohort**  **CVC N=57** |  |
| **Time of observation**  **Median in months (range)** | | 24.4 (0.5-75.7) | 32.8 (0.3-53.1) | 47.5 (0.5-75.7) | 33.4 (0.3-53.1) |  |
| **Age**  **Median in years (range)** | | 68 (35-94) | 65.5 (35-86) | 70.5 (35-94) | 66 (38-86) |  |
| **Sex** | **Male** | 35 | 37 | 17 | 36 |  |
|  | **Female** | 41 | 21 | 19 | 21 |  |
| **UICC** | **I** | 14 | 17 | 9 | 17 |  |
|  | **II** | 17 | 24 | 7 | 23 |  |
|  | **III** | 22 | 17 | 12 | 17 |  |
|  | **IV** | 23 | / | 8 | / |  |
| **T stage** | **1** | 11 | 4 | 4 | 4 |  |
|  | **2** | 12 | 15 | 7 | 15 |  |
|  | **3** | 42 | 37 | 17 | 36 |  |
|  | **4** | 11 | 2 | 8 | 2 |  |
| **N stage** | **0** | 39 | 41 | 18 | 40 |  |
|  | **N1-N3** | 37 | 17 | 18 | 17 |  |

**Supplementary Table 4** – Clinicopathological information on the M0 patients with colorectal cancer from the DU- and HE-cohorts where successful surgical resection of the tumour margins was achieved (R0) and from whom samples were collected intraoperatively from the draining vein (DV) and/or the central venous catheter (CVC).

|  |  | **DV=111 samples** | | **CVC = 85 samples** | |
| --- | --- | --- | --- | --- | --- |
|  |  | **DU-cohort**  **DV N=53** | **HE-cohort**  **DV N=58** | **DU-cohort**  **CVC N=28** | **HE-cohort**  **CVC N=57** |
| **Time of observation**  **Median in months (range)** | | 27.2 (0.8-75.7) | 32.8 (0.3-53.1) | 48.6 (0.8-75.7) | 33.4 (0.3-53.1) |
| **Age**  **Median in years (range)** | | 68 (35-94) | 65.5 (35-86) | 74.5 (35-94) | 66 (38-86) |
| **Sex** | **Male** | 22 | 37 | 13 | 36 |
|  | **Female** | 31 | 21 | 15 | 21 |
| **UICC** | **I** | 14 | 17 | 9 | 17 |
|  | **II** | 17 | 24 | 7 | 23 |
|  | **III** | 22 | 17 | 12 | 17 |
| **T stage** | **1** | 7 | 4 | 4 | 4 |
|  | **2** | 11 | 15 | 7 | 15 |
|  | **3** | 31 | 37 | 14 | 36 |
|  | **4** | 4 | 2 | 3 | 2 |
| **N stage** | **0** | 31 | 41 | 16 | 40 |
|  | **N1-N3** | 22 | 17 | 12 | 17 |
| **Neo-adjuvant therapy** | **No** | 45 | 40 | 24 | 39 |
|  | **Yes** | 8 | 18 | 4 | 18 |
| **Adjuvant therapy** | **No** | NA | 44 | NA | 43 |
|  | **Yes** | NA (~22) | 14 | NA (~12) | 14 |

DV - draining vein; CVC - central venous catheter; UICC - Union for International Cancer Control

**Supplementary Table 5** - Parameters used for enumeration of circulating tumour cells (CTCs), tumour-derived extracellular vesicle (tdEVs), white blood cells (WBCs), lymphocyte-derived extracellular (ldEVs) and bare nuclei using ACCEPT tool and R script.

|  | Channel (Marker) | Parameter | CTC | tdEV | WBC | ldEV | Bare Nucleus |
| --- | --- | --- | --- | --- | --- | --- | --- |
| **ACCEPT-UT*** | DAPI (DNA) | Mean Intensity | >45 | ≤5 | >30 | n.a. | >30 |
|  | DAPI (DNA) | Max Intensity | n.a. | n.a. | >50 | n.a. | n.a. |
|  | DAPI (DNA) | Size | n.a. | n.a. | >16 | n.a. | n.a. |
|  | DAPI (DNA) | Standard Deviation | n.a. | n.a. | n.a. | ≤5 | n.a. |
|  | PE (CK) | Mean Intensity | >60 | >60 | n.a. | n.a. | ≤5 |
|  | PE (CK) | Max Intensity | n.a. | >90 | n.a. | n.a. | n.a. |
|  | PE (CK) | Size | >16 and ≤400 | ≤150 | n.a. | n.a. | n.a. |
|  | PE (CK) | Overlay with DNA | >0.2 | n.a. | n.a. | n.a. | n.a. |
|  | PE (CK) | Perimeter | n.a. | >5 | n.a. | n.a. | n.a. |
|  | PE (CK) | P2A | n.a. | ≤1 | n.a. | n.a. | n.a. |
|  | PE (CK) | Eccentricity | n.a. | ≤0.8 | n.a. | n.a. | n.a. |
|  | PE (CK) | Standard Deviation | n.a. | n.a. | ≤5 | ≤5 | n.a. |
|  | APC (CD45) | Mean Intensity | ≤5 | ≤5 | >30 | >30 | ≤5 |
|  | APC (CD45) | Max Intensity | n.a. | n.a. | >50 | >50 | n.a. |
|  | APC (CD45) | Perimeter | n.a. | n.a. | n.a. | >5 | n.a. |
|  | APC (CD45) | Size | n.a. | n.a. | n.a. | ≤150 | n.a. |
|  | APC (CD45) | Eccentricity | n.a. | n.a. | n.a. | ≤0.85 | n.a. |
|  | DiOC (Empty) 1 | Mean Intensity | ≤5 | ≤5 | ≤5 | ≤5 | ≤5 |
|  | PerCP (Empty) 2 | Mean Intensity | ≤5 | ≤5 | ≤5 | ≤5 | ≤5 |
| **ACCEPT-DU**  Additional criteria | PE (CK) (in ACCEPT) | Eccentricity | <0.9 | n.a. | n.a. | n.a. | n.a. |
|  | CK / DNA (R script) | Size | CK > DNA | n.a. | n.a. | n.a. | n.a. |
|  | CK / DNA (R script) | Mean Intensity | CK > DNA or CK >150 | n.a. | n.a. | n.a. | n.a. |

Notes: *Values for CTCs and tdEVs were from Nanou2018 and values for WBCs and ldEVs were from Nanou2019; CTC - circulating tumour cells; tdEV - tumour-derived extracellular vesicle; WBC - white blood cell; ldEV - lymphocyte-derived extracellular vesicle

**Supplementary Figure 1**

**A**

| **Group** | **Samples** | **Settings** | **Detection** | **Median (mean;range)** | **Sens.** | **Spec.** | **C.K.** | **S. C** **.** | **S.C. p-value** |
| --- | --- | --- | --- | --- | --- | --- | --- | --- | --- |
| CRC+Othermalignant tumors | 362 | ACCEPT-DU | 26.24% | 0 (8.7348; 0-1790) | 0.65 | 0.92 | 0.59 | 0.68 | <0.0001 |
|  |  | ACCEPT-UT | 52.21% | 1 (18.7818; 0-3334) | 0.86 | 0.64 | 0.43 | 0.59 | <0.0001 |
|  |  | Manual | 32.04% | 0 (32.0635; 0-8284) | / | / | / | / | / |
| Benign | 33 | ACCEPT-DU | 12.12% | 0 (0.1212; 0-1) | 0.20 | 0.89 | 0.10 | 0.09 | 1 |
|  |  | ACCEPT-UT | 27.27% | 0 (0.7879; 0-11) | 0.60 | 0.79 | 0.29 | 0.38 | 0 |
|  |  | Manual | 15.15% | 0 (0.1818; 0-2) | / | / | / | / | / |
| Healthy | 93 | ACCEPT-DU | 12.9% | 0 (0.1505; 0-2) | 0.67 | 0.89 | 0.23 | 0.31 | 0.0027 |
|  |  | ACCEPT-UT | 32.26% | 0 (0.6667; 0-20) | 1.00 | 0.70 | 0.13 | 0.32 | 0.0018 |
|  |  | Manual | 3.23% | 0 (0.0323; 0-1) | / | / | / | / | / |

Notes: Detection - Detection rate; Sens. - Sensitivity; Spec. - Specificity; C.K. - Cohens Kappa; S. C. - Spearman rank order correlation coefficient; S. C. p-value - Spearman rank order correlation p-value

**B**

|  | | **ACCEPT-DU** | | **Chi^2^** | **Chi^2^**  **(Yates corr)** |  |  |  | **ACCEPT-UT** | | **Chi^2^** | **Chi^2^**  **(Yates corr)** |
| --- | --- | --- | --- | --- | --- | --- | --- | --- | --- | --- | --- | --- |
|  |  | **Neg** | **Pos** |  |  |  |  |  | **Neg** | **Pos** |  |  |
| **Manual counting** | **Neg** | 226 | 20 | 130.12 | 127.22 |  | **Manual counting** | **Neg** | 157 | 89 | 79.07 | 77.07 |
|  | **Pos** | 41 | 75 |  |  |  |  | **Pos** | 16 | 100 |  |  |


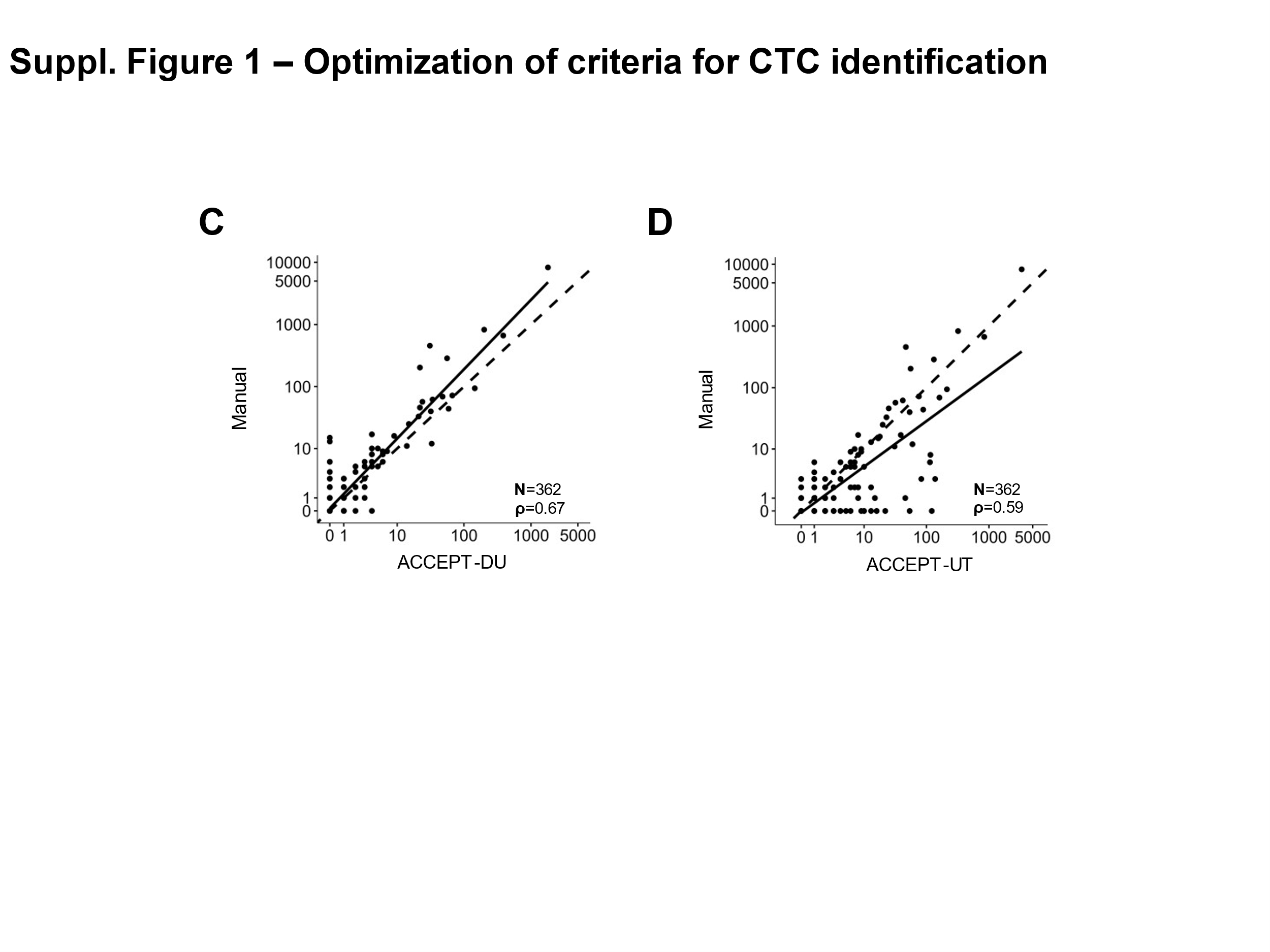


**Suppl. Figure 1 – Validation of the ACCEPT-DU gates. (A)** CellSearch-CTC counts obtained with ACCEPT software using the settings previously described (ACCEPT-UT), the new settings (ACCEPT-DU) and upon manual enumeration (Manual). Considered were 362 R0 CRC (DV/CVC) and other malignant tumours from the DU-cohort, the 33 benign from the same cohort, and the 93 samples from healthy donors of the IMMC06 cohort. Statistics for ACCEPT-DU and ACCEPT-UT were done using the manual counts as reference. **(B)** Sample positivity and negativity determined with the manual settings and with the ACCEPT-DU and ACCEPT-UT settings in the 362 samples from patients with malignant tumours as in (A). **(C)** Counts obtained manually and with the ACCEPT-DU settings for the complete collective of 362 samples as in (A). **(D)** Counts obtained manually and with the ACCEPT-UT settings for the complete collective of 362 samples as in (A).

**Supplementary Figure 2**

**
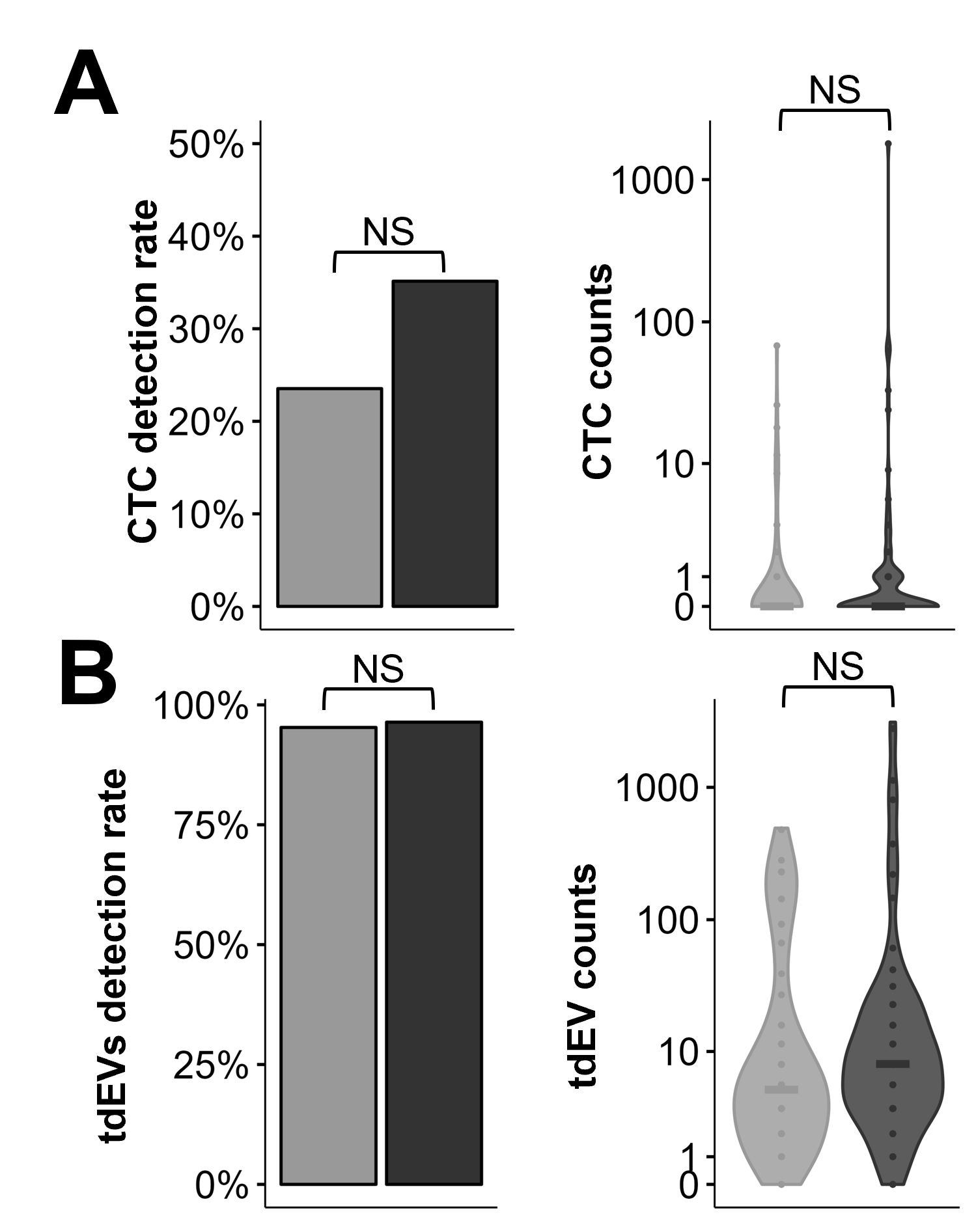
**

**Suppl. Figure 2 – Counts and detection rate of CTCs and tdEVs (A)** Detection rates (positivity rates) and counts of CTCs and **(B)** tdEVs in samples from CRC R0 M0 patients from the combined DU+HE cohort (N=85 CVCs; N=111 DVs). The horizontal lines represent the median.

**Supplementary Figure 3**

**
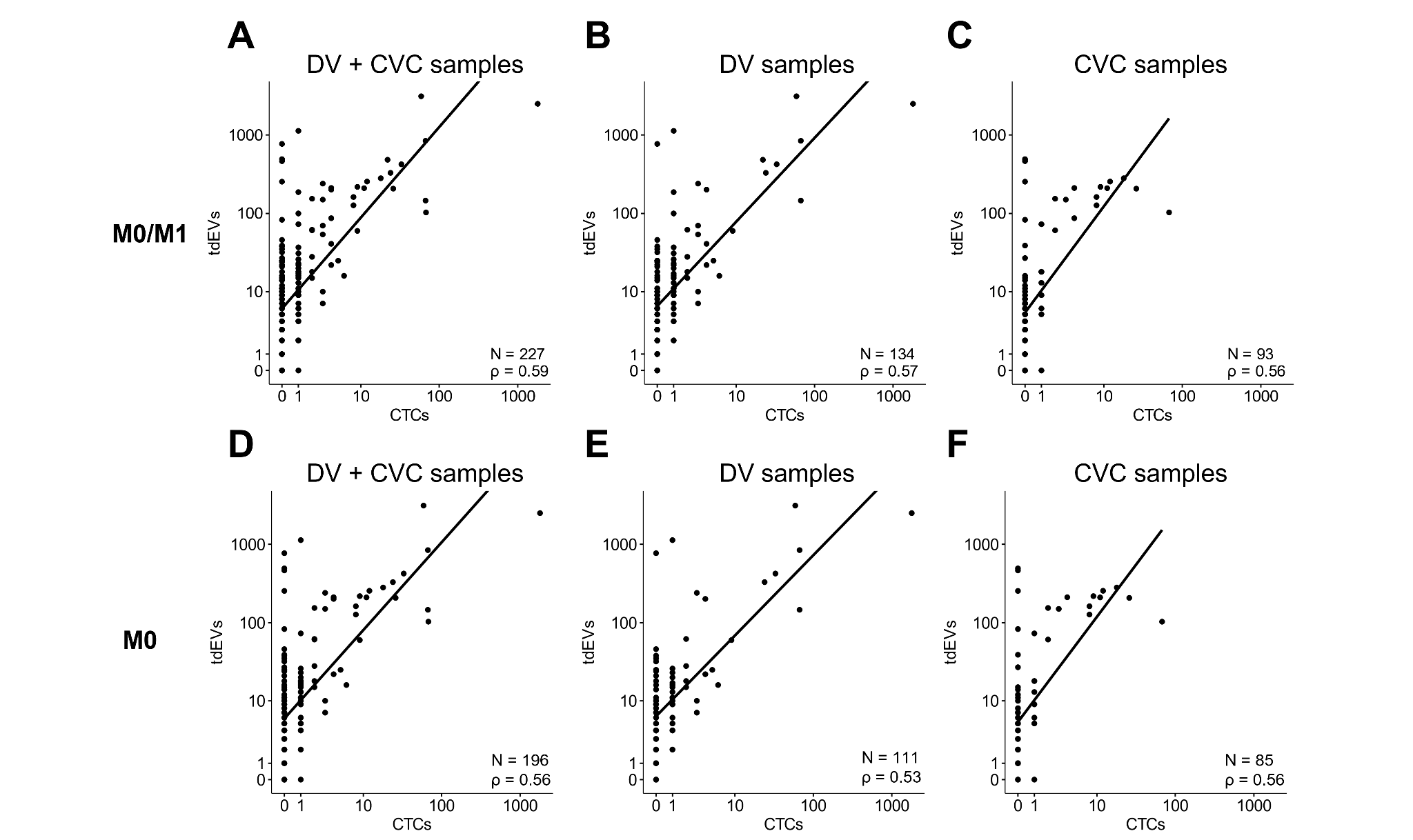
**

**Suppl. Figure 3 – Correlation between CTCs and tdEVs in samples from colorectal R0 patients from the DU+HE cohort. (A+D)** Draining vein (DV) and central venous catheter (CVC) samples (M0/M1 N = 227, p <0.0001; M0 N = 196, p <0.0001). **(B+E)** DV samples (M0/M1 N = 134, p <0.0001; M0 N = 111, p <0.0001). **(C+F)** CVC samples (N = 93, p <0.0001; M0 N = 85, p <0.0001).

**Supplementary Figure 4**


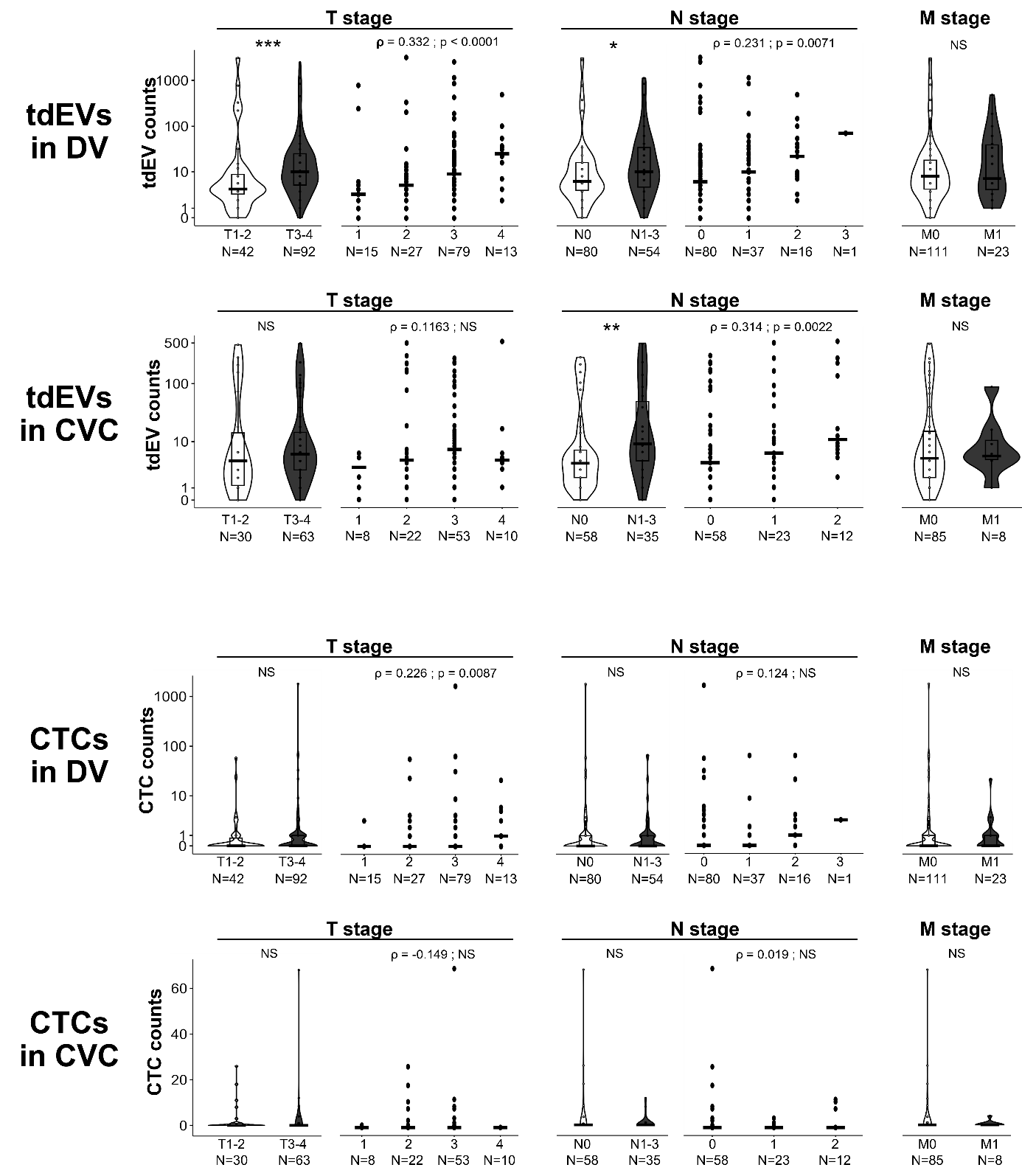


**Suppl. Figure 4 – Association between tdEVs and CTCs detected in the draining vein (DV) and central venous catheter (CVC), and clinicopathological features.** Particle counts in the collective of patients with colorectal cancer where successful surgical resection of the tumour margins was achieved (R0) and their association with pT-, M- and pN-staging of the respective patients. The statistics for grouped stages were performed with Mann-Whitney U testing while the statistics for individual stages were performed with Spearman correlation analyses.

**Supplementary Figure 5**

**
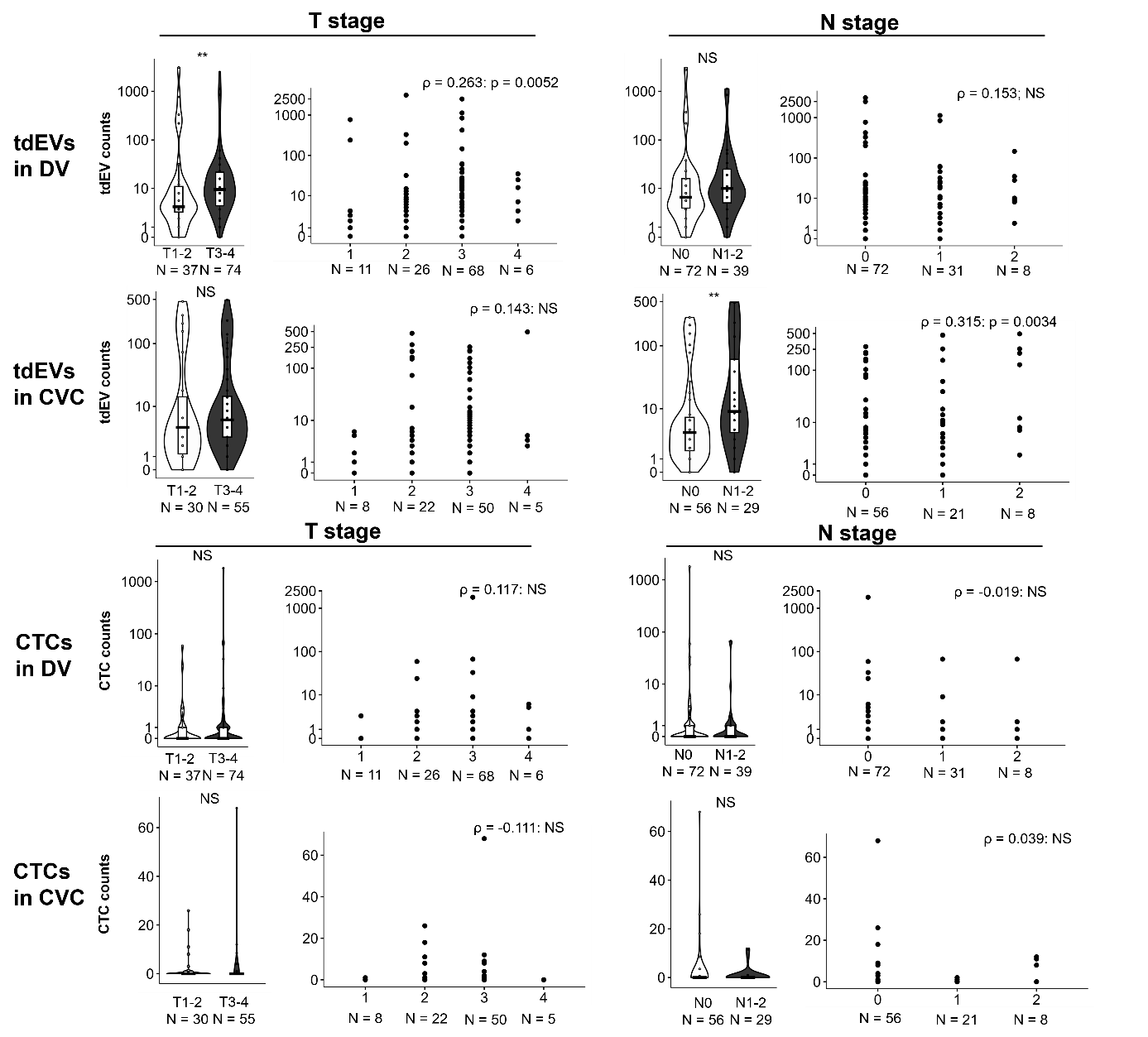
**

**Suppl. Figure 5 – Association between tdEVs and CTCs detected in the draining vein (DV) and central venous catheter (CVC), and clinicopathological features of M0 patients.** Particle counts in the collective of patients with M0 colorectal cancer where successful surgical resection of the tumour margins was achieved (R0) and their association with pT- and pN-, stages of the respective patients. The statistics for grouped stages were performed with Mann-Whitney U testing while the statistics for individual stages were performed with Spearman correlation analyses.

**Supplementary Figure 6**

**
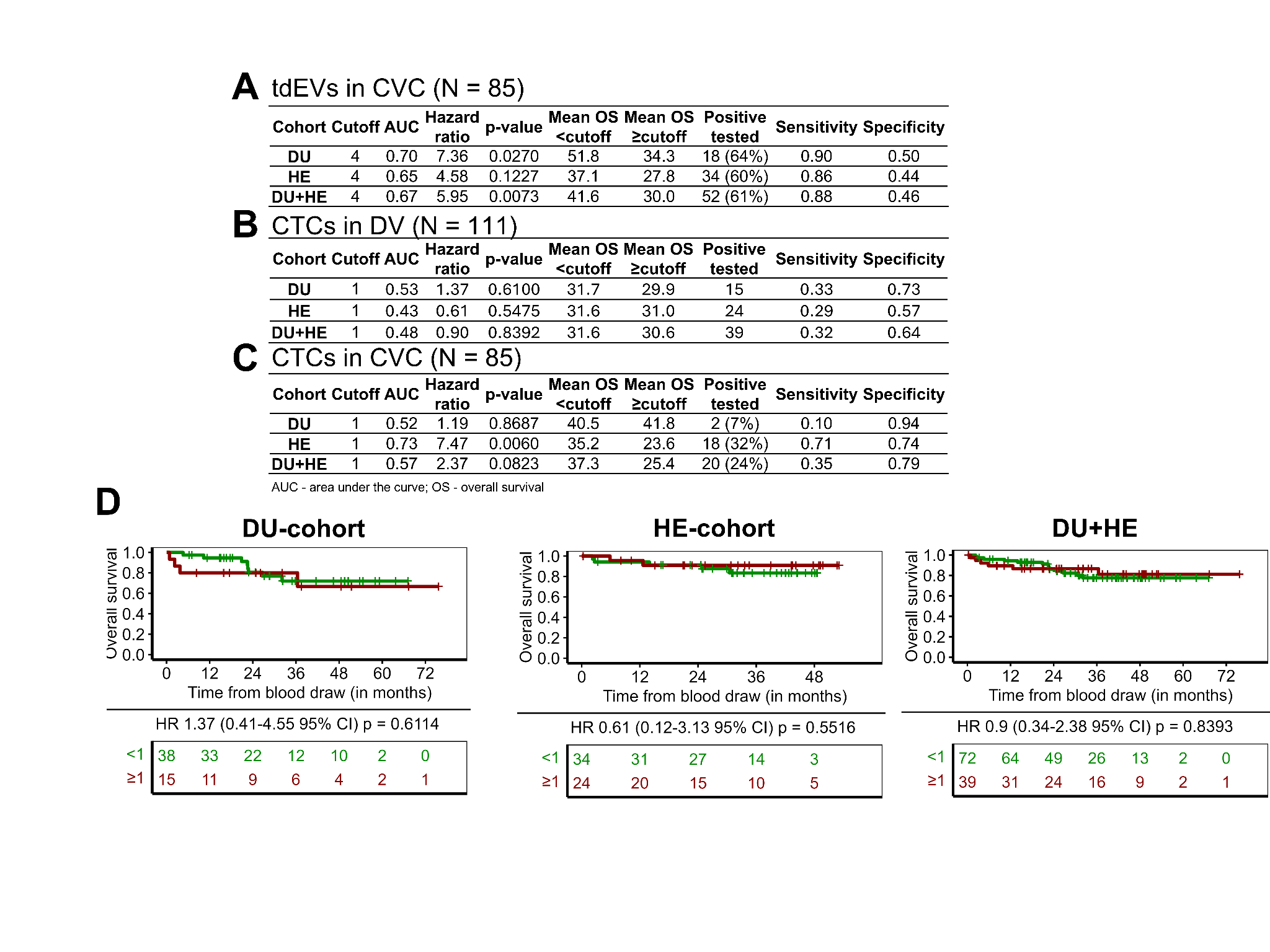
**

**Suppl. Figure 6 – CTC and tdEV cutoffs of M0 patients. (A)** Most relevant cutoffs for tdEV counts in CVC samples of M0 patients from the DU-cohort (N=28) and validation on the HE (N=57) and DU+HE cohorts (N=85). **(B)** Most relevant cutoffs for CTC counts detected in DV samples of M0 patients from the DU-cohort (N=53) and validation on the HE (N=58) and DU+HE cohorts (N=111). **(C)** Most relevant cutoffs defined for CTC counts detected in CVC samples of M0 patients from the DU-cohort (N=28) and validation on the HE (N=57) and DU+HE cohorts (N=85). **(D)** Kaplan-Meier estimates of overall survival for patients dichotomized based on the absence or presence (≥1 CTC cutoff) in DV samples of the DU- (N=53), HE- (N=58), and DU+HE (N=111) cohorts of M0 patients.

**Supplementary Figure 7**

**
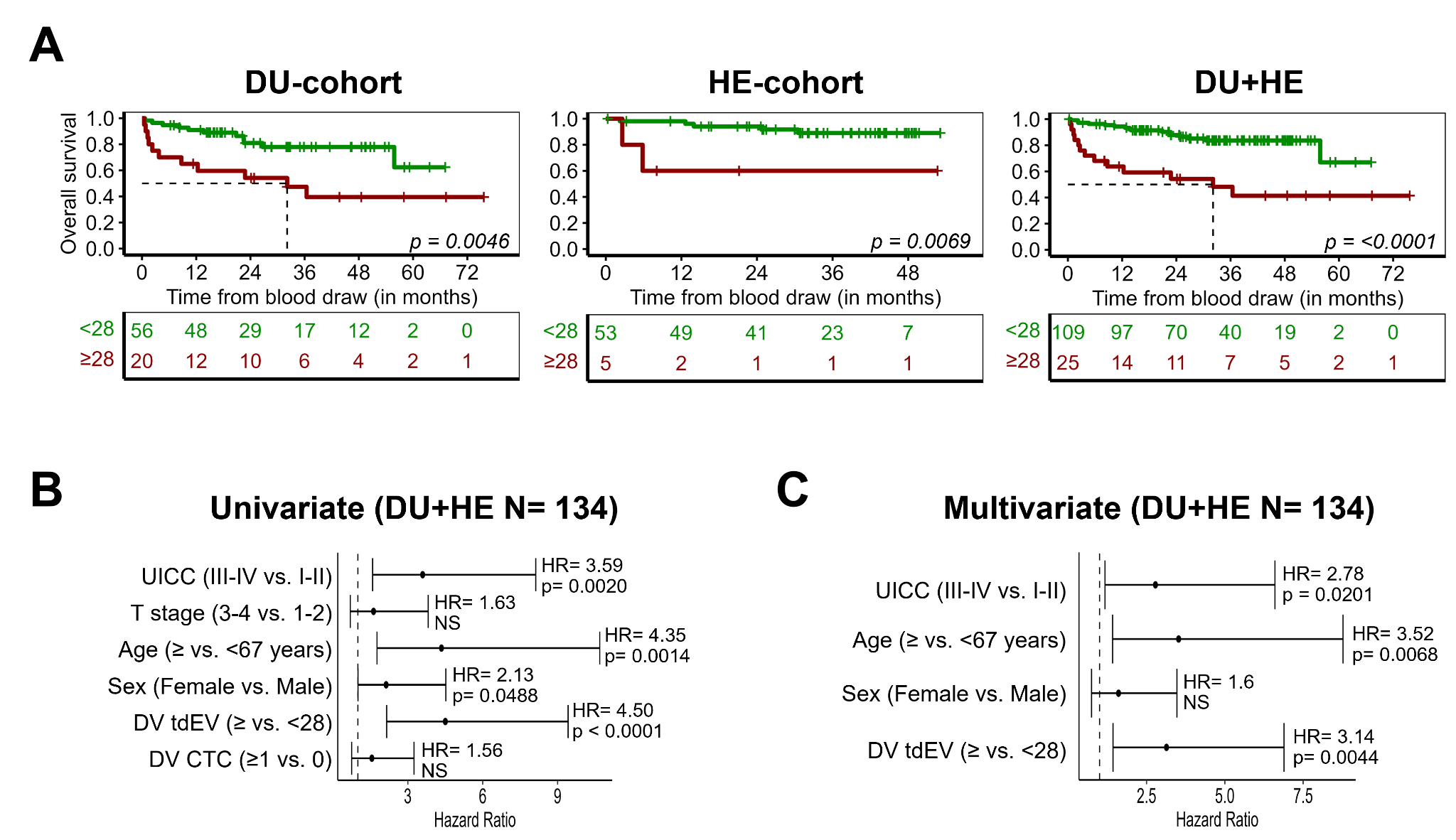
**

**Suppl. Figure 7 – Prognostic value of CS-tdEVs detected in DV samples of CRC R0 patients.** (**A**) Kaplan-Meier estimates of overall survival for patients dichotomized on the basis of the 28 tdEV cut-off in the DU (N=76), HE (N=58), and DU+HE (N=134) cohorts of patients. (**B**) Univariate analysis of clinicopathological factors (including the ≥28 tdEV cut-off) in the DU+HE cohort of patients (N=134). (**C**) Multivariate analysis of clinicopathological factors, including the ≥28 tdEV cut-off, in the DU+HE cohort of patients (N=134).

**Supplementary Figure 8**

**
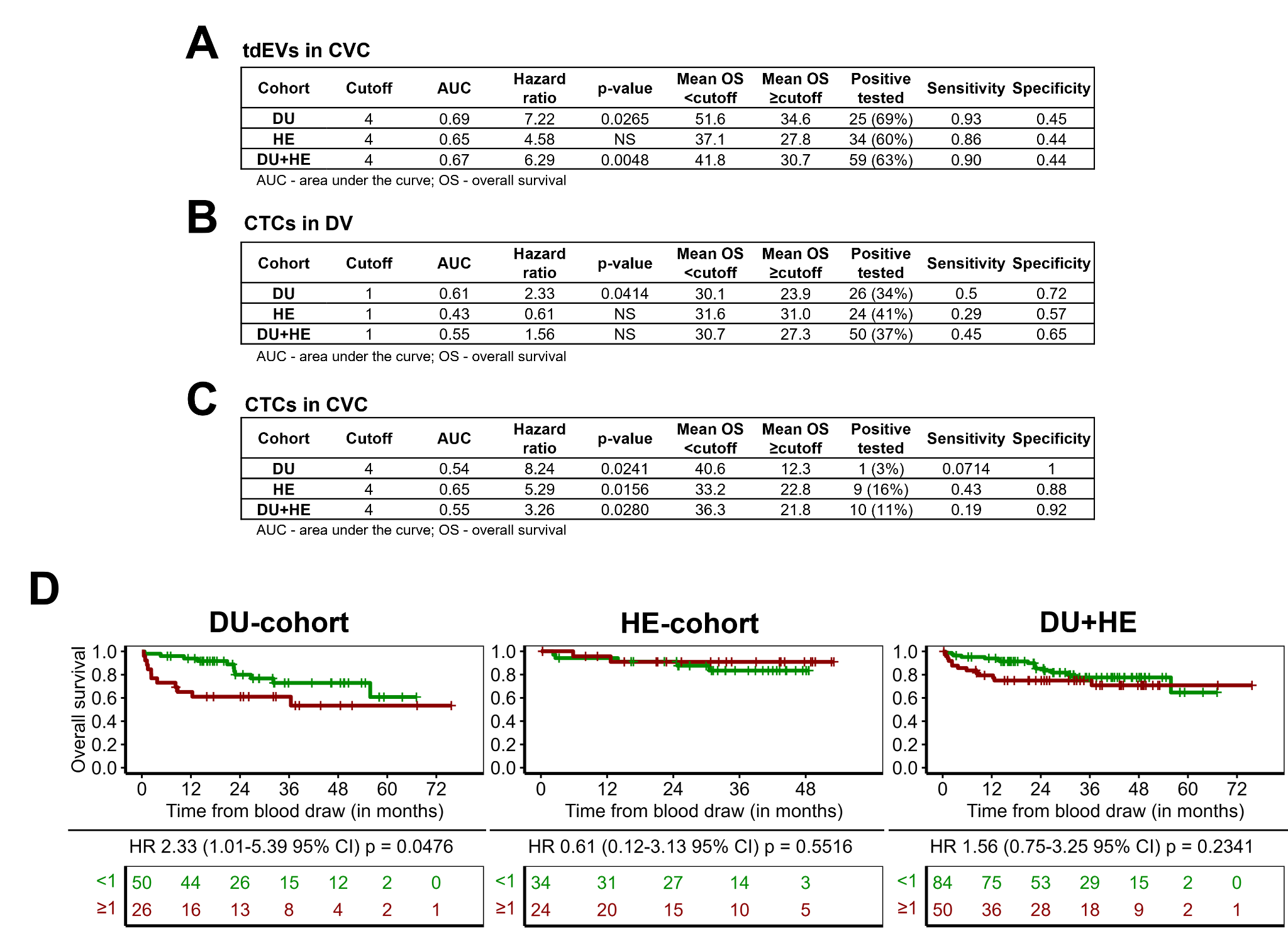
**

**Suppl. Figure 8 – CTC and tdEV cutoffs. (A)** Most relevant cutoffs for tdEV counts in CVC samples of patients from the DU-cohort (N=36) and validation on the HE (N=57) and DU+HE cohorts (N=93). **(B)** Most relevant cutoffs for CTC counts detected in DV samples of patients from the DU-cohort (N=76) and validation on the HE (N=58) and DU+HE cohorts (N=134). **(C)** Most relevant cutoffs defined for CTC counts detected in CVC samples of patients from the DU-cohort (N=36) and validation on the HE (N=57) and DU+HE cohorts (N=93). (**D**) Kaplan-Meier estimates of overall survival for patients dichotomized based on the absence or presence (≥1 CTC cutoff) in DV samples of the DU- (N=76), HE- (N=58), and DU+HE (N=134) cohorts of patients.

**Supplementary Figure 9**


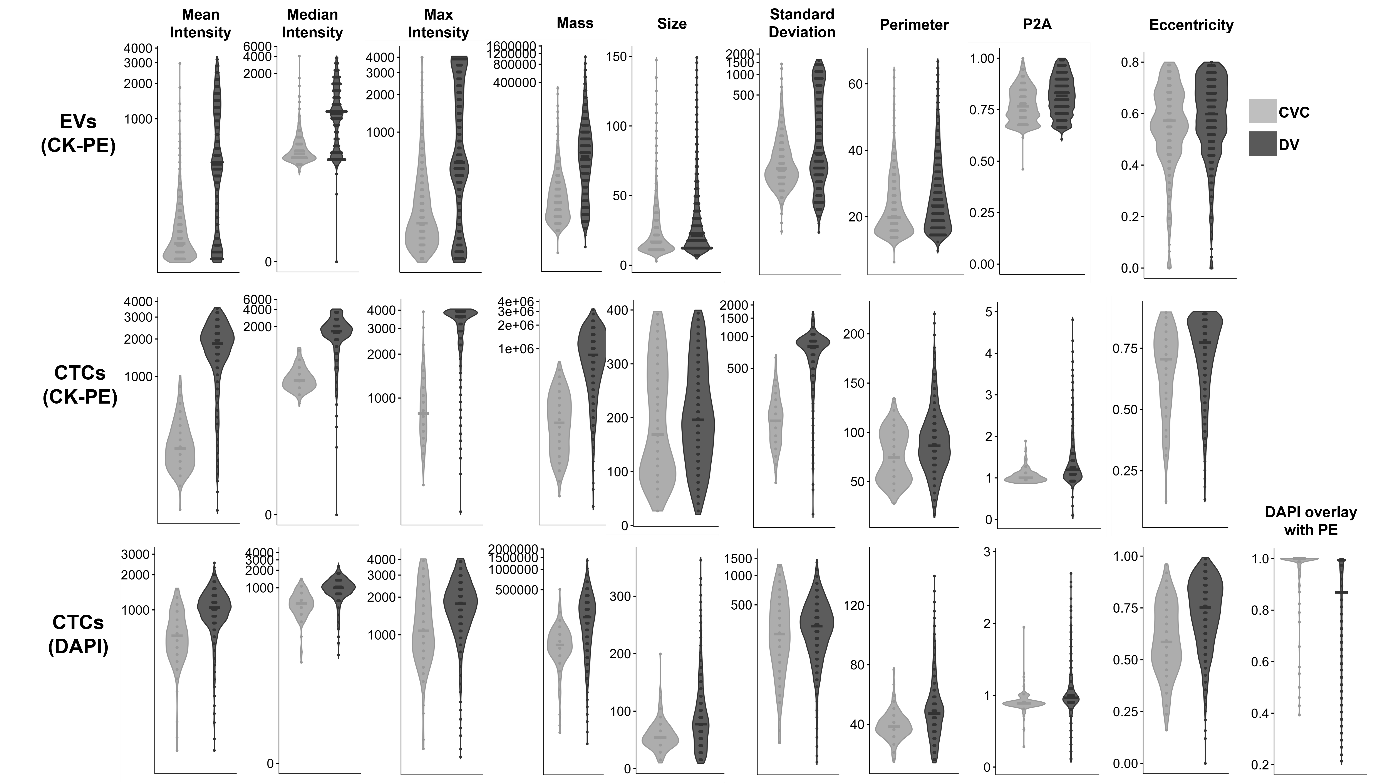


**Suppl. Figure 9 – Phenotypic characterization of tdEVs and CTCs in draining vein (DV) and central venous catheter (CVC) samples of M0 R0 patients.** Quantification by ACCEPT of nine parameters extracted from the Phycoerythrin (PE) signal detected in tdEVs and CTCs, as well as nine parameters extracted from the DAPI signal in CTCs. In addition, there is also the overlay of DAPI and PE signals determined with R-script based on the ACCEPT data. For analysis were considered R0 M0 CRC patients of the DU+HE cohort (DV= 111 samples, CVC= 85 samples) containing N=10756 DV-tdEVs; N=3883 CVC-tdEVs; N=2106 DV-CTCs; N=179 CVC-CTCs. Statistical tests to compare the quantified parameters between the CVC and DV were highly significant (p <0.0001) aside from the comparison of DAPI standard deviation for CTCs (p <0.05).

**Supplementary Figure 10**


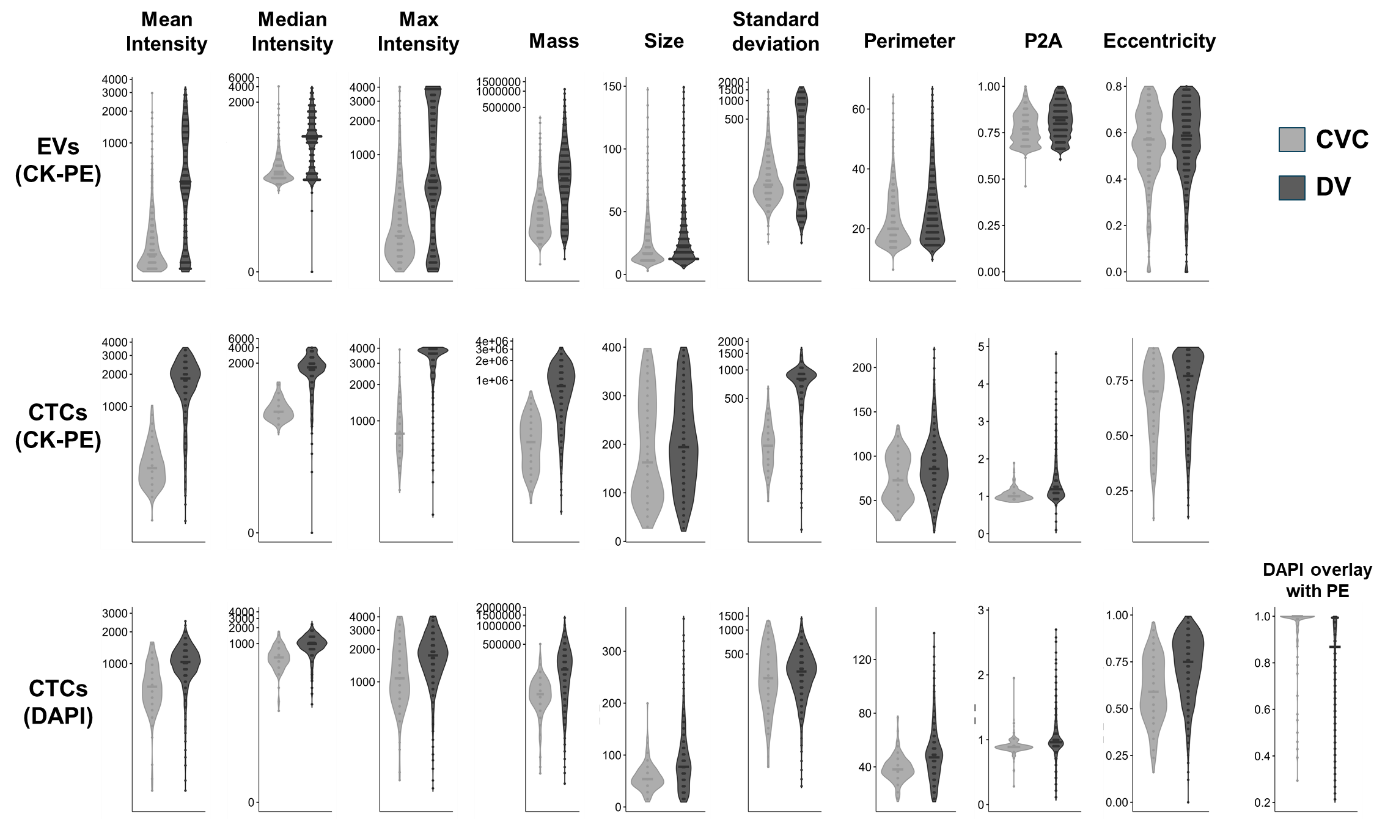


**Suppl. Figure 10 – Phenotypic characterization of tdEVs and CTCs in draining vein (DV) and central venous catheter (CVC) samples.** Quantification by ACCEPT of nine parameters extracted from the Phycoerythrin (PE) signal detected in tdEVs and CTCs, as well as nine parameters extracted from the DAPI signal in CTCs. In addition, there is also the overlay of DAPI and PE signals determined with R-script based on the ACCEPT data. For analysis were considered R0 CRC patients of the DU+HE cohort (DV= 134 samples, CVC= 93 samples) containing N=11866 DV-tdEVs; N=4016 CVC-tdEVs; N=2145 DV-CTCs; N=183 CVC-CTCs. Statistical tests to compare the quantified parameters between the CVC and DV were highly significant (p <0.0001) aside from two comparisons of PE size and DAPI standard deviation for CTCs (p <0.05).

**Supplementary Figure 11**


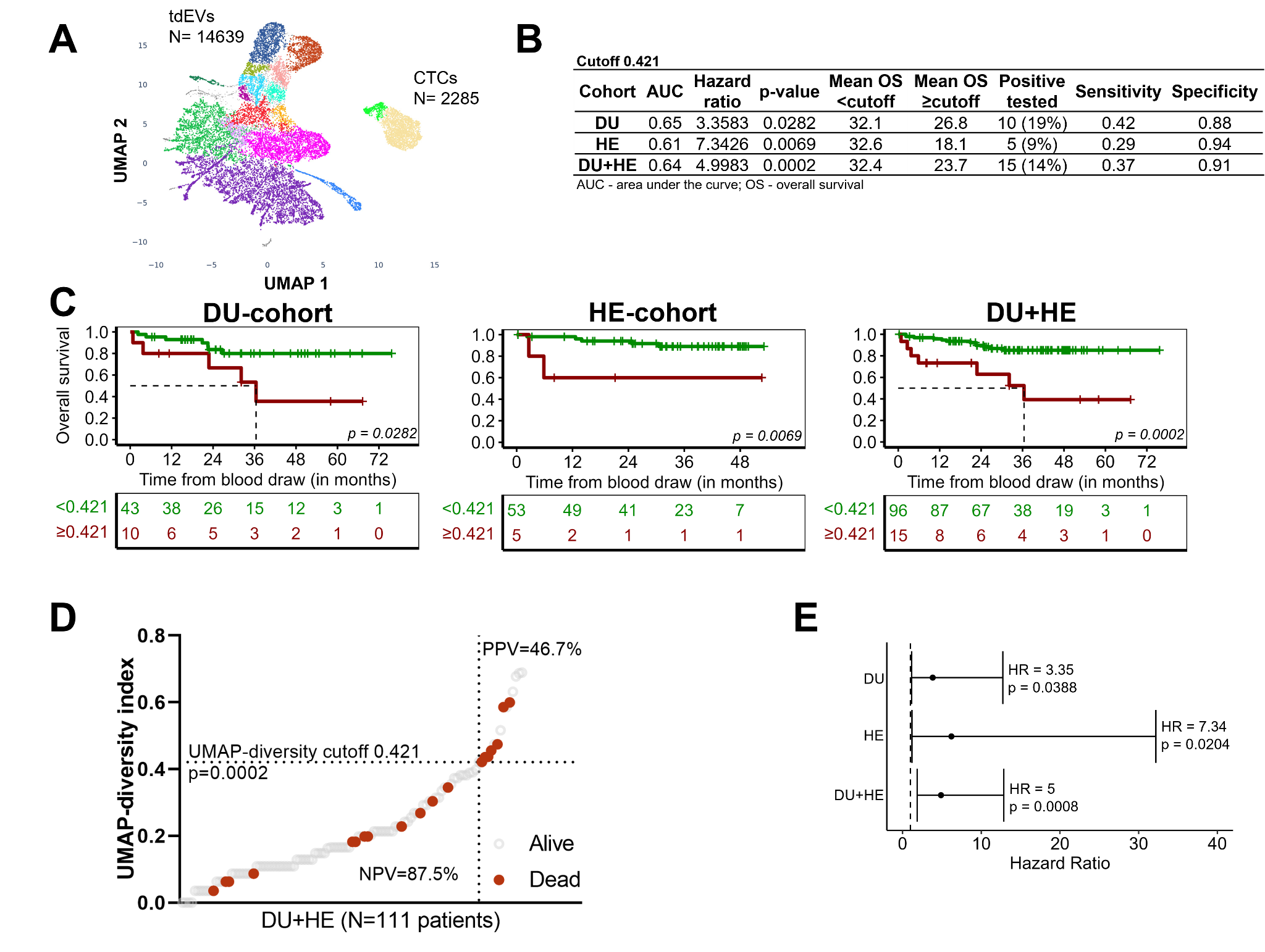


**Suppl. Figure 11 – Shannon diversity calculated for CTCs and tdEVs found in the draining vein samples of M0 patients. (A)** UMAP distribution of tdEVs and CTCs found in the DU+HE cohort, and the 18 clusters defined according to the UMAP clustering tool. **(B)** Most relevant Shannon diversity index cutoff. **(C)** Kaplan-Meier estimates of overall survival (OS) for patients dichotomized based on the Shannon diversity cutoff of 0.4214 in the DU (N=53), HE (N=58), and DU+HE (N=111) cohorts of patients. **(D)** Shannon diversity index calculated for each of the patients of the DU+HE cohort (N=111) showing the positive predictive value (PPV) and negative predictive value (NPV) of the cutoff 0.4214 as a biomarker. In red are indicated the patients that died. **(E)** Hazard ratio (HR) of patients with Shannon diversity index ≥0.4214.

**Supplementary Figure 12**

**
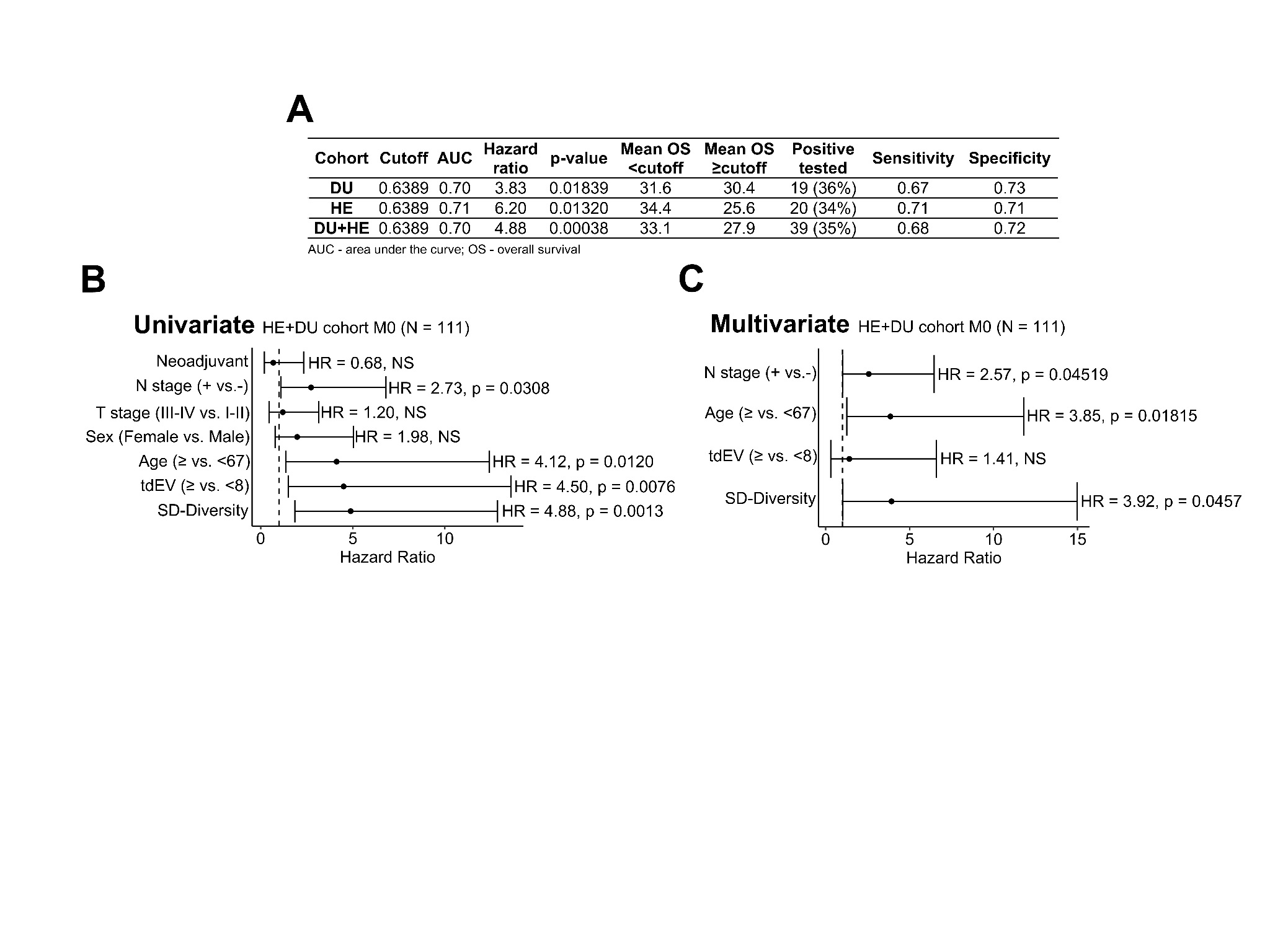
**

**Suppl. Figure 12 – SD-diversity calculated for CTCs and tdEVs found in the DV samples of R0 M0 patients. (A)** Most relevant SD-diversity index cutoff tested in the DU, HE and DU+HE cohorts. **(B)** Association of clinicopathological features, tdEVs and SD-diversity (≥0.6389) with the survival of patients from the DU+HE-cohort (N=111) by univariate analysis. **(C)** Multivariate analysis including DV tdEV count and SD-diversity in the model of patients from the DU+HE-cohort (N=111).

**Supplementary Figure 13**

**
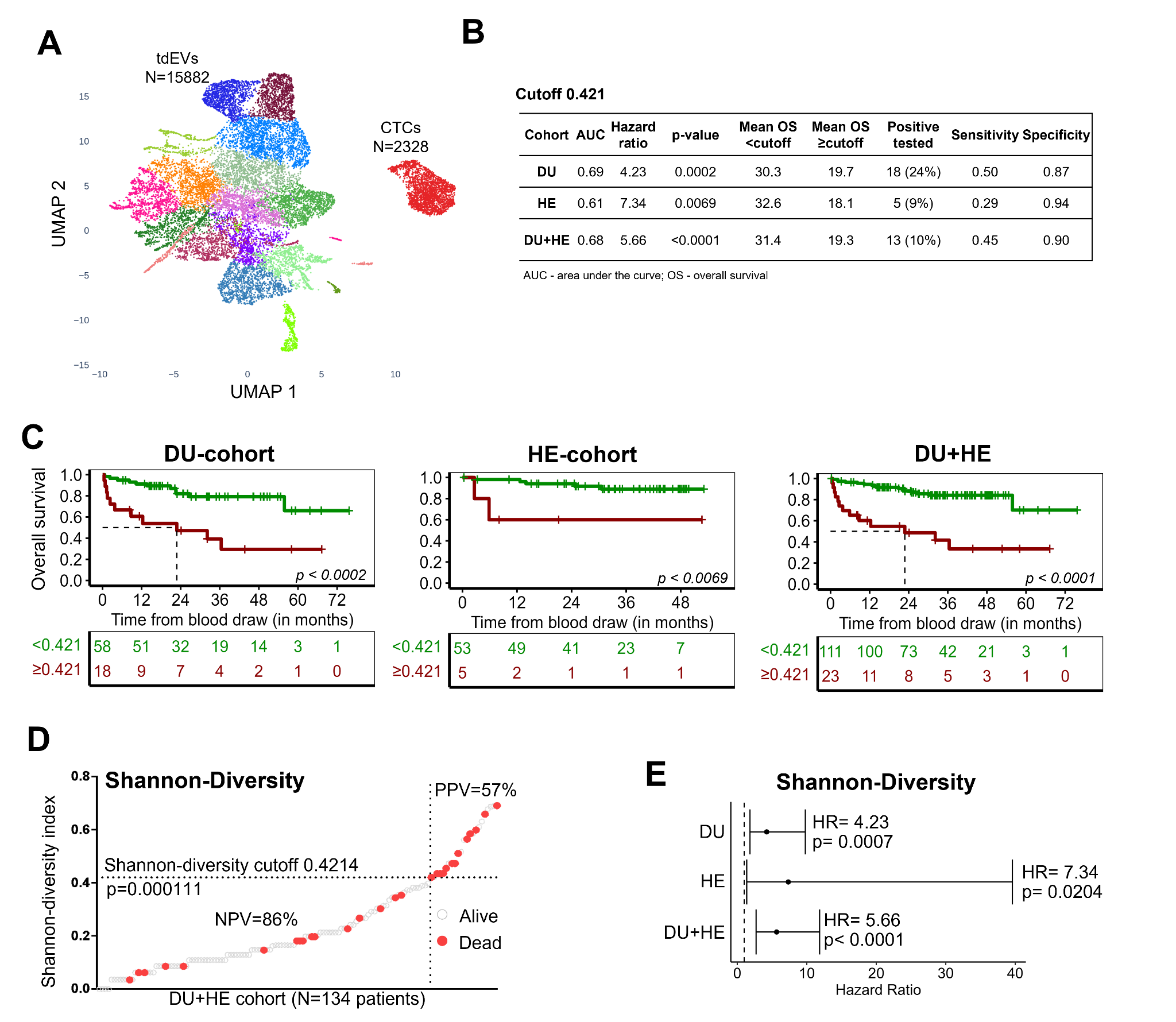
**

**Suppl. Figure 13 – Shannon diversity calculated for CTCs and tdEVs found in the draining vein (DV) samples (UICC I-IV).** (**A**) UMAP distribution of tdEVs and CTCs found in the DU+HE cohort, and the 18 clusters defined according to the UMAP clustering tool. (**B**) Most relevant Shannon diversity index cutoff. (**C**) Kaplan-Meier estimates of OS for patients dichotomized based on the Shannon diversity cutoff of 0.4214 in the DU (N=76), HE (N=58), and DU+HE (N=134) cohorts of patients. (**D**) Shannon diversity index calculated for each of the patients of the DU+HE cohort (N=134) showing the positive predictive value (PPV) and negative predictive value (NPV) of the cutoff 0.4214 as a biomarker. In red are indicated the patients that died. (**E**) Hazard ratio (HR) of patients with Shannon diversity index ≥0.4214.

**Supplementary Figure 14**


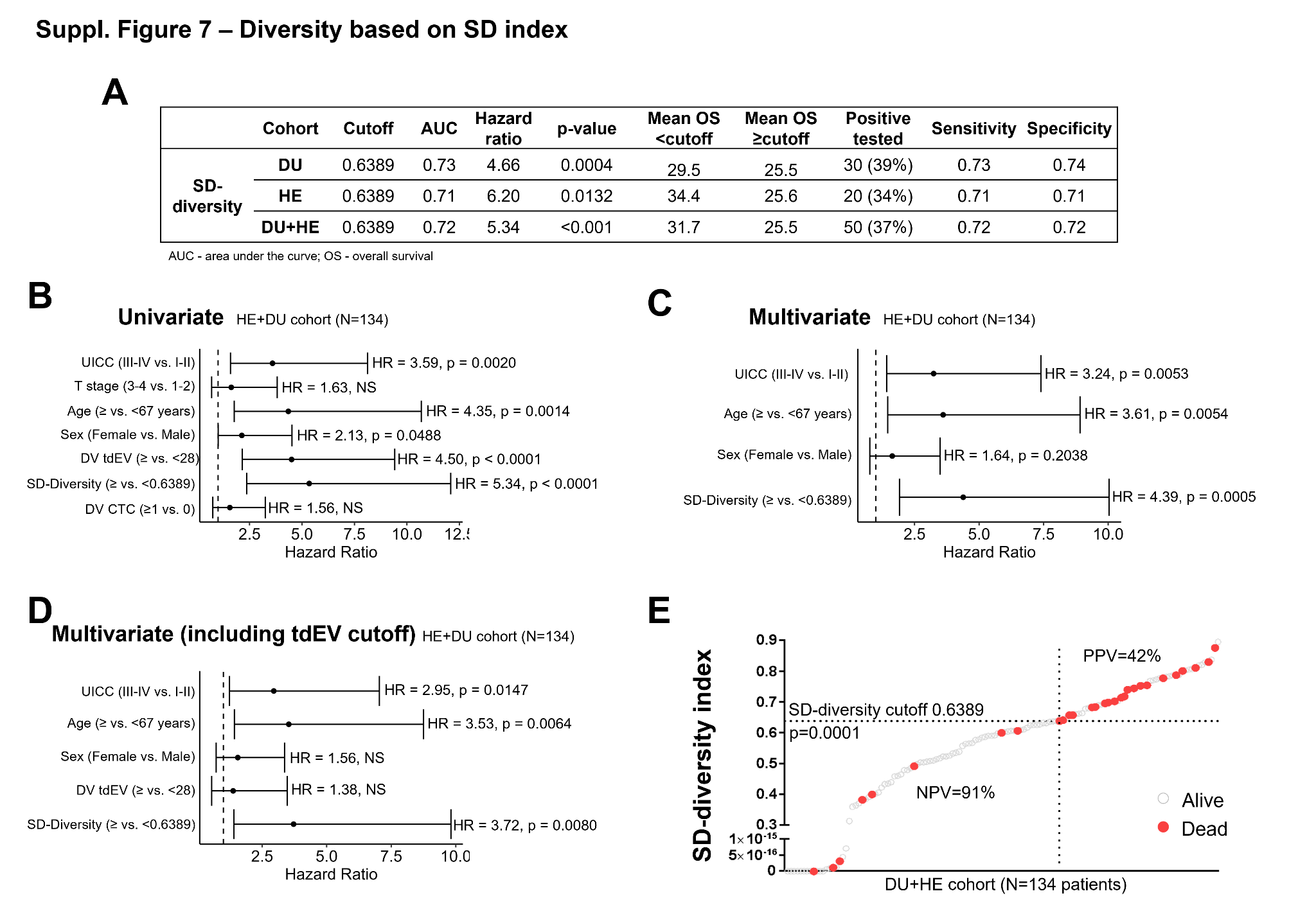


**Suppl. Figure 14 – SD-diversity calculated for CTCs and tdEVs found in the DV samples (UICC I-IV). (A)** Most relevant SD-diversity index cutoff tested in the DU, HE and DU+HE cohorts. **(B)** Association of clinicopathological features, tdEVs, CTCs and SD-diversity (≥0.6389) with the survival of patients from the DU+HE-cohort (N=134) by Univariate analysis. **(C)** Multivariate analysis excluding the DV tdEV count from the model. **(D)** Multivariate analysis including DV tdEV count and SD-diversity in the model. **(E)** SD-diversity index calculated for each of the N=134 patients of the DU+HE cohort showing the positive predictive value (PPV) and negative predictive value (NPV) of the cutoff 0.6389 as a biomarker. In red are indicated the patients that died.

**Supplementary Figure 15**

**
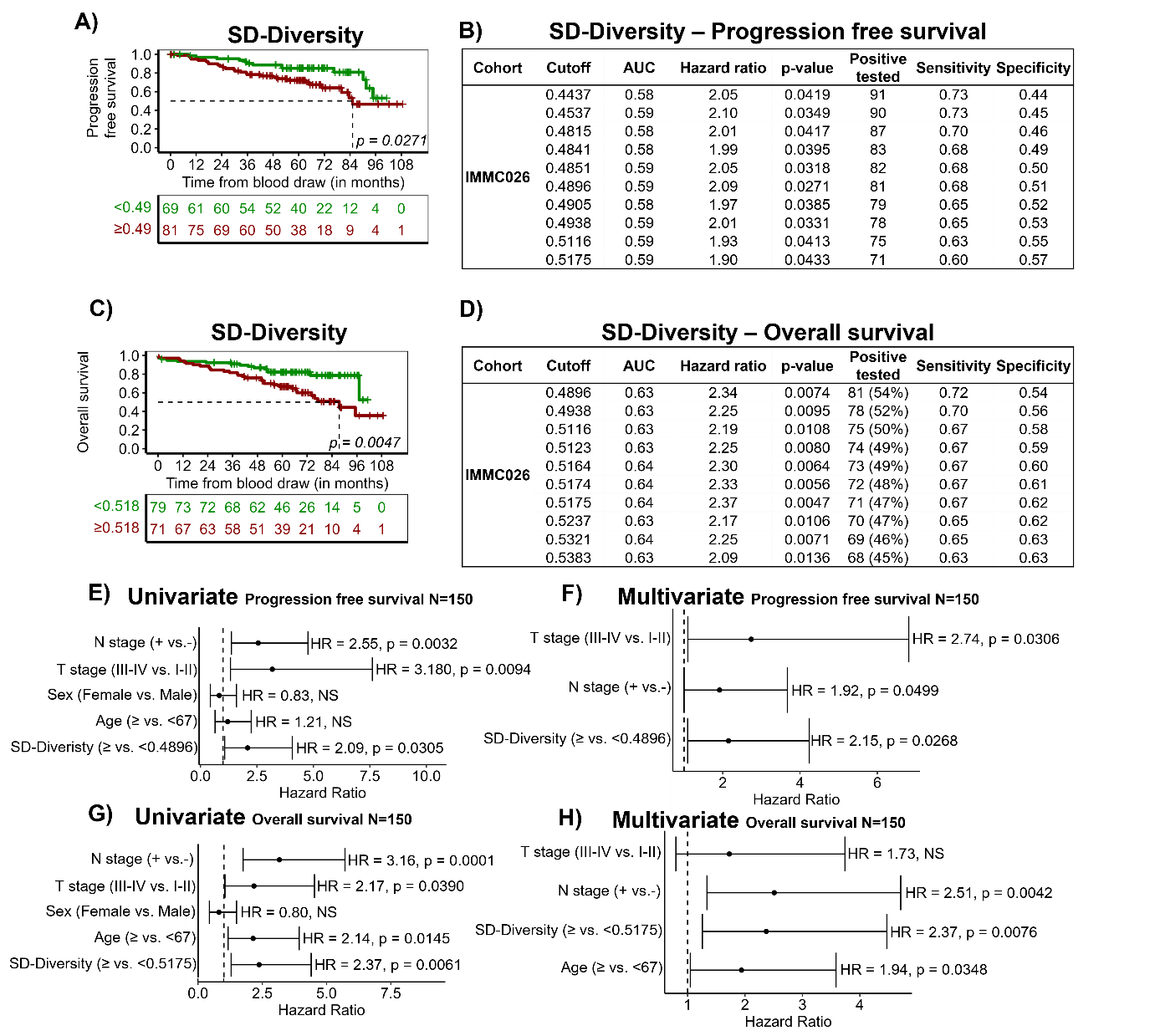
**

**Suppl. Figure 15 – SD-diversity calculated for CTCs and tdEVs found in preoperative samples of the IMMC-26**  **study. (A)** Kaplan-Meier estimate of progression-free survival (PFS) for patients dichotomized based on the SD-diversity diversity cutoff of 0.49 in the IMMC-26 cohort (N=150). **(B)** Most relevant SD-diversity cutoffs based on PFS.  **(C)** Kaplan-Meier estimate of overall survival for patients dichotomized based on the SD-diversity cutoff of 0.52 in the IMMC-26 cohort (N=150). **(D)** Most relevant SD-diversity cutoffs based on OS. **(E)** Association of clinicopathological features including the SD-diversity cutoff of 0.49 with PFS of patients from the IMMC-26 study. **(F)** Multivariate analysis including SD-diversity based on PFS. **(G)** Association of clinicopathological features including the SD-diversity cutoff of 0.49 with the OS of patients from the IMMC-26 study. **(H)** Multivariate analysis including SD-diversity based on OS.
